# Understanding the mechanism of Nb-MXene bioremediation with green microalgae

**DOI:** 10.1038/s41598-022-18154-3

**Published:** 2022-08-23

**Authors:** Michał Jakubczak, Dominika Bury, Muhammad Abiyyu Kenichi Purbayanto, Anna Wójcik, Dorota Moszczyńska, Kaitlyn Prenger, Michael Naguib, Agnieszka Maria Jastrzębska

**Affiliations:** 1 Faculty of Materials Science and Engineering, Warsaw University of Technology, Wołoska 141, 02-507 Warsaw, Poland; 2 Institute of Metallurgy and Materials Science, Polish Academy of Sciences, W. Reymonta 25, 30-059 Cracow, Poland; 3 Department of Physics and Engineering Physics, Tulane University, New Orleans, LA 70118 USA

**Keywords:** Two-dimensional materials, Environmental impact

## Abstract

Rapidly developing nanotechnologies and their integration in daily applications may threaten the natural environment. While green methods of decomposing organic pollutants have reached maturity, remediation of inorganic crystalline contaminants is major problem due to their low biotransformation susceptibility and the lack of understanding of material surface-organism interactions. Herein, we have used model inorganic 2D Nb-based MXenes coupled with a facile shape parameters analysis approach to track the mechanism of bioremediating 2D ceramic nanomaterials with green microalgae *Raphidocelis subcapitata*. We have found that microalgae decomposed the Nb-based MXenes due to surface-related physicochemical interactions. Initially, single and few-layered MXene nanoflakes attached to microalgae surfaces, which slightly reduced algal growth. But with prolonged surface interaction, the microalgae oxidized MXene nanoflakes and further decomposed them into NbO and Nb_2_O_5_. Since these oxides were nontoxic to microalgal cells, they consumed Nb-oxide nanoparticles by an uptake mechanism thus enabling further microalgae recovery after 72 h of water treatment. The uptake-associated nutritional effects were also reflected by cells’ increased size, smoothed shape and changed growth rates. Based on these findings, we conclude that short- and long-term presence of Nb-based MXenes in freshwater ecosystems might cause only negligible environmental effects. Notably, by using 2D nanomaterials as a model system, we show evidence of the possibility of tracking even fine material shape transformations. In general, this study answers an important fundamental question about the surface interaction-associated processes that drive the mechanism of 2D nanomaterials’ bioremediation as well as provides the fundamental basis for further short- and long-term investigations on the environmental effects of inorganic crystalline nanomaterials.

## Introduction

Nanomaterials have gained considerable interest since their discovery, and various nanotechnologies have recently reached the upscaling stage^1^. Unfortunately, the integration of nanomaterials in daily life applications may result in accidental spills stemming from inappropriate disposal, incautious management, or incomplete safety infrastructure. Therefore, it is reasonable to assume that nanomaterials, including two-dimensional (2D) nanomaterials, may be released into the natural environment where their behavior and bioactivity are not fully understood. Consequently, it is not surprising that ecotoxicity concerns focus on the possibility of 2D nanomaterials’ infiltration into water systems^2–6^. In these ecosystems, some 2D nanomaterials can interact with various organisms at different trophic levels, including microalgae.

Microalgae are primitive organisms, naturally occurring in freshwater and marine ecosystems, where they produce a wide range of chemical products via photosynthetic action^7^. Consequently, they are crucial in aquatic ecosystems^8–12^, but are also sensitive, low-cost, and widely used ecotoxicity indicators^13,14^. Since microalgae cells proliferate quickly and rapidly respond to the occurrence of different compounds, they show promise for the development of green approaches for water remediation of organic pollutants^15,16^.

Inorganic ions can be removed from water by algae cells via bioadsorption and accumulation^17,18^. Several algae species such as *Chlorella*, *Anabaena inaequalis*, *Westiellopsis prolifica*, *Stigeoclonium tenue* and *Synechococcus sp.* have been found to tolerate and even nourish themselves with toxic metal ions such as Fe^2+^, Cu^2+^, Zn^2+^ and Mn^2+^^19^. Other studies found that Cu^2+^, Cd^2+^, Ni^2+^, Zn^2+^, or Pb^2+^ ions limit the growth of *Scenedesmus* algae by changing cells’ morphology and disrupting their chloroplasts^20,21^.

Green methods of decomposing organic pollutants and removing heavy metal ions has attracted the attention of scientists and engineers around the world. This is primarily because these pollutants are easy to handle in the liquid phase. However, inorganic crystalline pollutants are characterized by low water solubility and a little susceptibility to various biotransformations, thus causing significant difficulties with remediation processes, with little progress in the field^22–26^. Consequently, discovering green solutions for remediating nanomaterials remains a challenging and underexplored field. As there is a high degree of uncertainty around the effects of 2D nanomaterials’ biotransformations, facile approaches for elucidating their possible decomposition pathways during remediation process are not yet available.

In this study, we have used green microalgae as an active agent for water bioremediation from inorganic ceramic materials, combined with in situ tracking the decomposition process of MXene as a representative of inorganic ceramic materials. The term 'MXene' reflects M_n+1_X_n_T_*x*_ stoichiometry of the material, in which M is an early transition metal, X is carbon and/or nitrogen, T_*x*_ represents surface terminations (e.g., –OH, –F, –Cl), and *n* = 1, 2, 3 or 4^27,28^. Since their discovery by Naguib et al.^27^ in 2011, many unique properties have been reported for MXenes, including electrical conductivity, hydrophilicity, and bioactivity, supporting their broad application potential in various fields, e.g., EMI shielding, micro-supercapacitors, biosensing, cancer treatment, and membrane filtration^27,29,30^. Furthermore, MXenes can be considered model 2D systems due to their excellent colloidal stability in combination with feasible biointeractions^31–36^.

**Figure 1:**
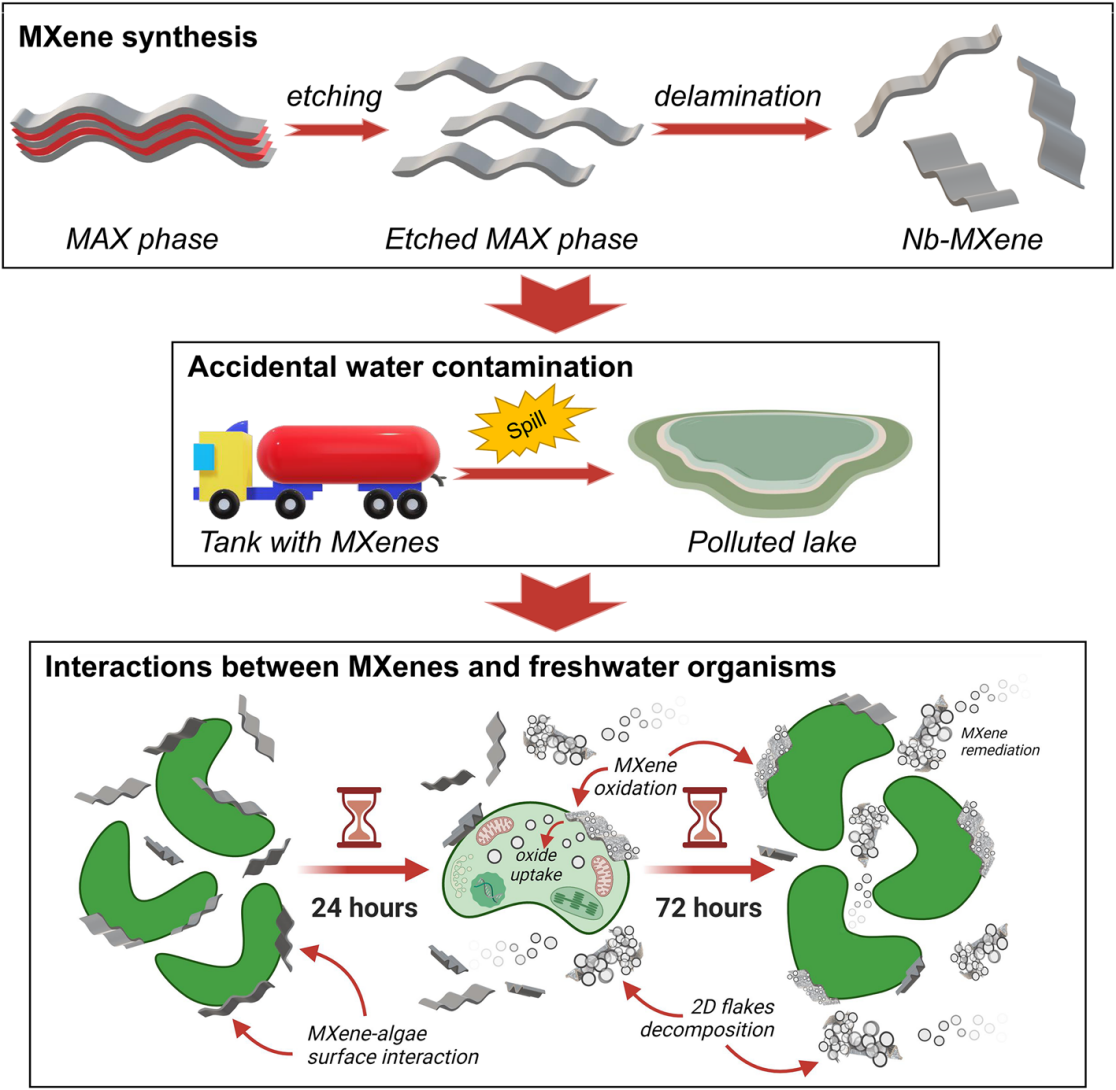
A research approach and evidence-based research hypothesis for the MXene remediation with green microalgae *Raphidocelis subcapitata*. Note that this is only a schematic representation of the evidence-based hypothesis. The lake environment differs from the used growth medium and conditions (e.g., day and night cycle and limitation of available essential nutrients). Created with BioRender.com.

Therefore, an approach developed in this paper and our research hypothesis is presented in Fig. 1. According to this hypothesis, the microalgae decompose Nb-based MXenes into nontoxic compounds due to surface-related physicochemical interactions, which enable further algae recovery. To test the hypothesis, we choose two niobium-based representatives of the early transition metal carbides and/or nitrides (MXenes) family—viz., Nb_2_CT_*x*_ and Nb_4_C_3_T_*x*_.

Consequently, by using MXene as the model system, we open a door for investigating various bioeffects that cannot be observed for other conventional nanomaterials. In particular, we prove the possibility of bioremediating 2D nanomaterials such as Nb-based MXenes by microalgae *Raphidocelis subcapitata*. Microalgae are able to decompose Nb-MXenes into nontoxic NbO and Nb_2_O_5_ oxides which is also contributed to their nutrition via niobium uptake mechanism. Overall, this study answers an important fundamental question about the surface physicochemical interaction-associated processes that drive the mechanism of 2D nanomaterials’ bioremediation. In addition, we have developed a facile shape parameters-based approach for tracking even fine transformations of 2D nanomaterials’ shape. This provides an inspiration for further short- and long-time investigations on various environmental effects of inorganic crystalline nanomaterials. Therefore, our study increases the understanding of material surface-organism interactions. We also give the fundamental basis for extended short- and long-time investigations of their possible effects in freshwater ecosystems that can be now verified by the simple way.

## Results and discussion

### Preparation and characterization of model Nb-MXenes

MXenes are an interesting family of materials possessing unique and attractive physical and chemical properties, which leads to many potential applications. These properties strongly depend on their stoichiometry and surface chemistry. Therefore, in our research, we investigated two types of delaminated single-layered (SL) Nb-based MXenes, Nb_2_CT_*x*_ and Nb_4_C_3_T_*x*_, as distinct bioeffects could be observed for those nanomaterials. MXenes are obtained from their parental material via top-down selective etching of atomically thin A layers from MAX phases. MAX phases are ternary ceramics consisting of transition metal carbide blocks “glued” together with thin layers of “A” elements, such as Al, Si, and Sn, with the stoichiometry M_n_AX_n−1_. The morphology of the parent MAX phases was observed with a scanning electron microscopy (SEM) and agreed with the previous studies^37^ (see Supplementary Information, SI, Fig. S1). After removal of the Al layer with 48% HF (hydrofluoric acid) to produce multilayered (ML) Nb-MXene. The morphology for both ML-Nb_2_CT_*x*_ and ML-Nb_4_C_3_T_*x*_ was investigated with scanning electron microscopy (SEM) (Figs. S1c and S1d, respectively) and the typical lamellar MXene morphology resembling 2D nanoflakes separated from each other by elongated slit-shaped pores was observed. Both Nb-MXenes show many similarities with MXene phases previously synthesized via acidic etching^27,38^. After confirming the MXene structure, we moved to delamination by intercalation of tetrabutylammonium hydroxide (TBAOH), followed by washing and sonication, after which we obtained the single-to-few layer (SL) 2D Nb-MXene nanoflakes.

**Figure 2:**
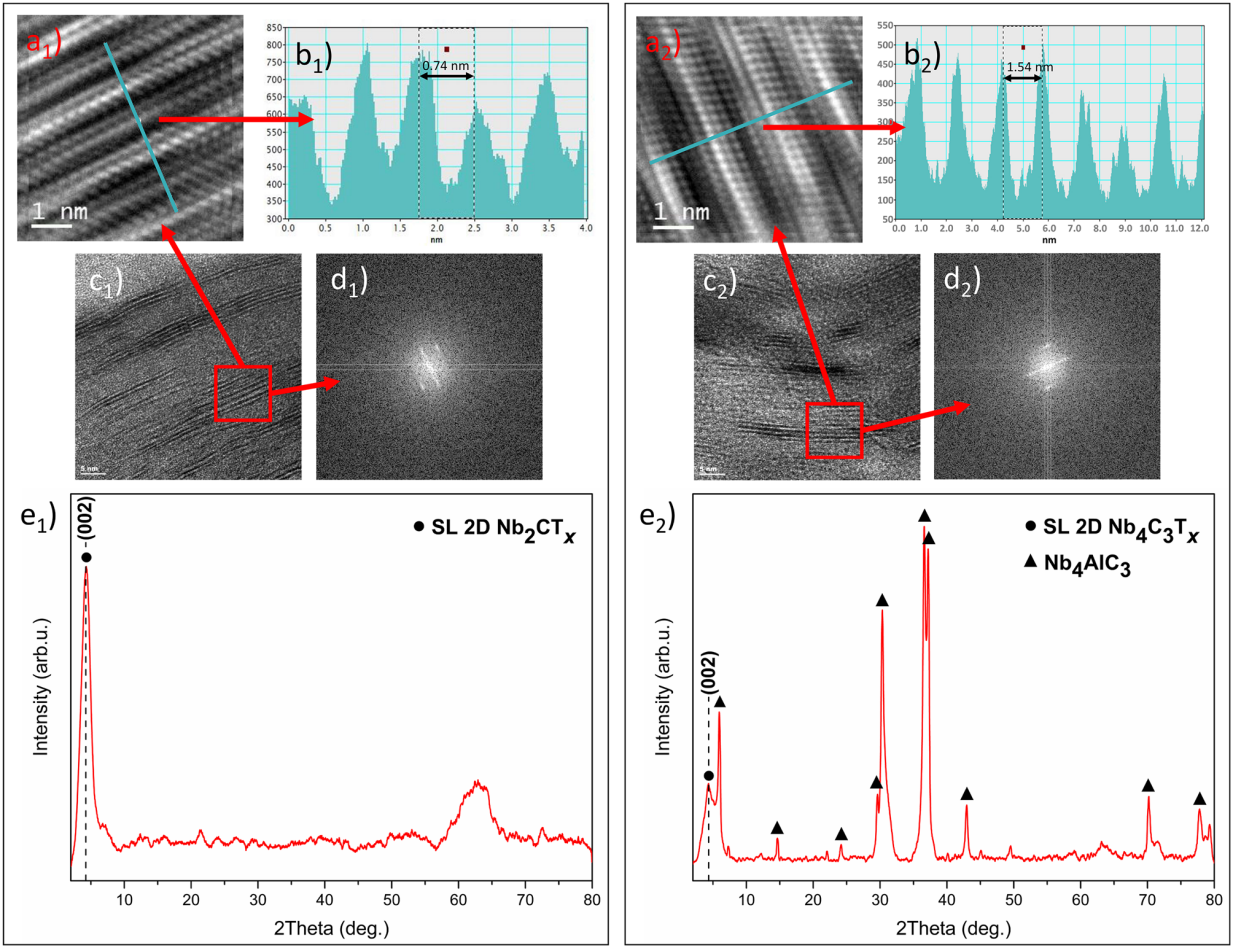
Characterization of SL Nb_2_CT_*x*_ and Nb_4_C_3_T_*x*_ MXene nanoflakes including (**a**) high-resolution electron microscopy (HRTEM) imaging of edge-viewed 2D nanoflake together with the corresponding, (**b**) intensity pattern, (**c**) inverse fast Fourier transform (IFFT), (**d**) fast Fourier transform (FFT), (**e**) XRD patterns obtained for Nb-MXenes. For SL 2D Nb_2_CT_*x*_, figures are denoted as (**a**_**1**_, **b**_**1**_, **c**_**1**_, **d**_**1**_, **e**_**1**_). For SL 2D Nb_4_C_3_T_*x*_ figures are denoted as (**a**_**2**_, **b**_**2**_, **c**_**2**_, **d**_**2**_, **e**_**1**_).

To check the efficiency of etching and further delamination, we utilized high-resolution transmission electron microscopy (HRTEM) and X-ray diffraction (XRD). The results from HRTEM processed with inverse fast Fourier transform (IFFT) and fast Fourier transform (FFT) imaging are shown in Fig. 2. The Nb-MXene nanoflakes were oriented edge-on to enable verifying the atomically layered structure and measure interplanar distances. The HRTEM images for Nb_2_CT_*x*_ and Nb_4_C_3_T_*x*_ MXene nanoflakes showed their atomically thin layered nature (see Fig. 2a_1_, a_2_), as previously reported by Naguib et al.^27^ and Jastrzębska et al.^38^. For two adjacent single layers of Nb_2_CT_*x*_ and Nb_4_C_3_T_*x*_, we determined the interlayer spacings to be 0.74 and 1.54 nm, respectively (Fig. 2b_1_, b_2_), which also agreed with our previous results^38^. This was additionally confirmed by inverse fast Fourier transform (Fig. 2c_1_, c_2_) and Fast Fourier transform (Fig. 2d_1_, d_2_) imaging, which revealed the spacing between single layers of Nb_2_CT_*x*_ and Nb_4_C_3_T_*x*_. The images show alternating light and dark bands corresponding to niobium and carbon atoms, which confirmed the layered pattern of investigated MXenes. Importantly, energy-dispersive X-ray spectroscopy (EDX) spectra obtained for both Nb_2_CT_*x*_ and Nb_4_C_3_T_*x*_ (Figs. S2a and S2b) suggest no residues of parental MAX phases, as peaks of Al were not detected.

XRD measurements for both SL Nb_2_CT_*x*_ and Nb_4_C_3_T_*x*_ MXenes, as shown in Fig. 2e_1_, e_2_, respectively. The (002) peaks positioned at 4.31 and 4.32 correspond to previously reported delaminated Nb_2_CT_*x*_, and Nb_4_C_3_T_*x*_ MXenes, respectively^38–41^. The XRD results also suggest some residual ML structures and MAX phase, but mostly for SL Nb_4_C_3_T_*x*_-related XRD pattern (Fig. 2e_2_). Stronger MAX peaks may be explained by the presence of minor MAX phase particles as compared to randomly stacked Nb_4_C_3_T_*x*_ layers.

### Elucidating the nontoxic concentrations for Nb-MXenes

Further study was concentrated on green microalgae belonging to the *R. subcapitata* species. We choose the microalgae because they are essential producers^42^ participating in the critical food web. They are also one of the best toxicity indicators due to the possibility of removing toxic agents that are transferred towards higher levels of the trophic chain^43^. Moreover, investigations on *R. subcapitata* could reveal the incidental toxicity of SL Nb-MXenes towards freshwater microorganisms in general. To elucidate this, researchers base the assumption that each microorganism has an individual sensitivity towards the presence of toxic compounds in the environment. For most organisms, minor concentrations of materials will not affect their growth, while concentrations above certain limits may inhibit them or even cause death. Therefore, for our studies concerning surface interactions between microalgae and MXenes and associated remediation, we started at verifying the harmless and toxic concentrations for Nb-MXenes. For this purpose, we tested 0 (as a reference), 0.01, 0.1, and 10 mg L^−1^ MXene concentrations and additionally challenged microalgae with very high MXene concentration (100 mg L^−1^ of MXene), likely an extreme and lethal value for any bioenvironment.

**Figure 3:**
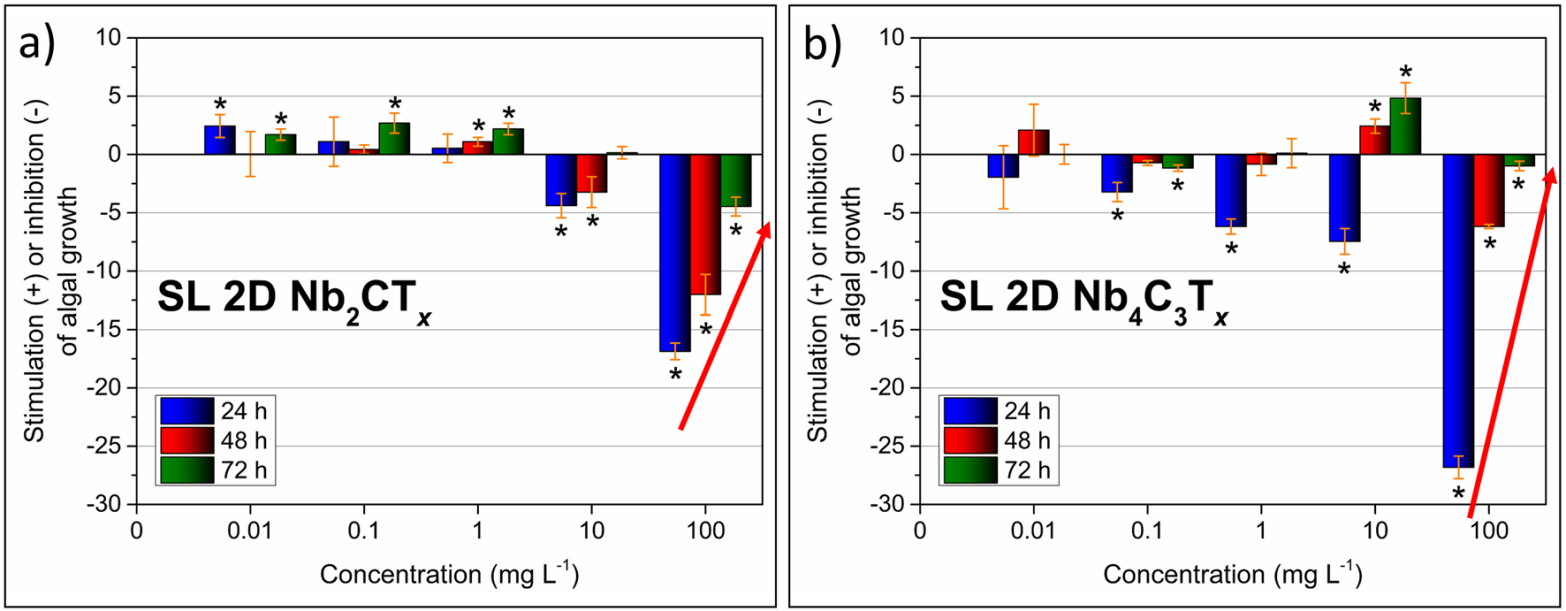
Stimulation (+) or inhibition (−) of microalgal growth in the presence of SL (**a**) Nb_2_CT_*x*_ and (**b**) Nb_4_C_3_T_*x*_ MXenes. The analysis was performed for 24, 48 and 72 h of MXene-microalgae interaction. Significant data (*t*-test, *p* < 0.05) were marked with an asterisk (*). The red arrows indicate the reversal inhibition-to-stimulation effect.

The effects of SL Nb-MXenes on microalgae are presented in Fig. 3 as a percentage of growth stimulation (+) or inhibition (-) of that measured for the 0 mg L^−1^ sample. For comparison purposes, the Nb-MAX phases and ML Nb-MXenes were also tested, and the results are shown in SI (see Fig. S3). Obtained results confirmed an almost complete lack of toxicity for SL Nb-MXenes in low concentrations ranging from 0.01 up to 10 mg L^−1^ as indicated by Fig. 3a, b. In the case of Nb_2_CT_*x*_, we observed that in mentioned range, the ecotoxicity did not exceed 5%.

On the other hand, low concentrations of Nb_4_C_3_T_*x*_ seemed to be a little more toxic, while not crossing 7%. As expected, we noticed much higher toxicity of MXenes and growth inhibition of microalgae at 100 mg L^−1^ concentration. Interestingly, the nontoxic/toxic effects for all materials did not display the same trend and time-dependence as compared to MAX or ML samples (see SI for more details). While for MAX phases (see Fig. S3) the toxicity reached about 15–25% and increased over time, the reverse trend was observed for the case of SL Nb_2_CT_*x*_ and Nb_4_C_3_T_*x*_ MXenes. The microalgal growth inhibition was reduced with time. After 24 h it reached about 17% and after 72 h—it dropped to less than 5% (Fig. 3a, b, respectively).

More importantly, for SL Nb_4_C_3_T_*x*_, microalgal growth inhibition reached about 27% after 24 h, but after 72 h, it diminished to about 1%. Therefore, we have labeled the observed effect as reversal inhibition-to-stimulation and it was more intense for SL Nb_4_C_3_T_*x*_ MXene. The stimulation of microalgal growth was noticed much earlier for Nb_4_C_3_T_*x*_ (already at 10 mg L^−1^ for 24 h of interaction) than to SL Nb_2_CT_*x*_ MXene. The reversal inhibition-to-stimulation effect was also visualized well by biomass doubling rate curves (check Fig. S4 for details). So far, only Ti_3_C_2_T_*x*_ MXene was investigated regarding ecotoxicity with diverse results. It demonstrated no toxicity towards zebrafish embryos^44^ but moderate ecotoxicity towards *Desmodesmus quadricauda* microalgae and *Sorghum saccharatum* plants^45^. Other examples of specific action include a higher toxicity towards cancerous cell lines than normal ones^46,47^. One would assume that the test conditions could influence microalgae growth alternations, as observed in the presence of the Nb-MXenes. For example, the pH of the chloroplast stroma of about 8 is optimal for the RuBisCO enzyme to work efficiently. Therefore, the rate of photosynthesis is negatively affected when the pH value alternates^48,49^. Nevertheless, we have not observed significant changes in pH during the experiment (see SI, Fig. S5 for details). In general, the microalgae cultures with Nb-MXenes tended to slightly decrease the solution pH over time. However, such a decrease was similar to the pH change of the pure medium. In addition, the range of detected alternations was similar to the one measured for pure microalgae culture (reference sample). Therefore, we conclude that photosynthesis was not affected by the pH alterations, which occurred over time.

Moreover, the as-synthesized MXenes possess surface terminations (denoted T_*x*_). These are mostly –O, –F, and –OH functional groups. However, surface chemistry is directly related to the synthesis method^34^. It is also known that these groups are randomly distributed over the surfaces, which makes it more challenging to predict their impact on the MXenes properties^50^. One could argue that T_*x*_ can be a catalytic force for niobium oxidation under illumination. The surface functional groups indeed supply many anchored sites for the base photocatalyst to form heterojunctions^51^. However, the composition of growth medium does not provide an efficient photocatalyst (the detailed composition of the culture medium can be found in SI, Table S6). Moreover, any surface modification is also of high importance as MXene bioactivity may transform due to the post-delamination treatments, oxidation, chemical surface modification with organic and inorganic compounds^52–56^ or surface charge engineering^38^. Therefore, to check whether the niobium oxides have any connection with the instability of material in culturing medium, we performed zeta (ζ) potential studies in microalgae nutritive medium, and DI water (for comparison). Our results indicate the fair stability of SL Nb-MXenes (for MAX and ML results, see SI, Fig. S6). The SL MXenes showed ζ-potential of about −10 mV. In the case of SL Nb_2_CT_*x*_, the ζ-value was a little more negative than that for Nb_4_C_3_T_*x*_. Such changes of ζ-value may indicate the absorption of positively charged ions from culturing medium on the surface of negatively charged MXene nanoflakes^38^. The time-wise measurements of ζ-potential and conductivity (check Figs. S7 and S8 in SI for more details) performed for Nb-MXenes in culturing medium seem to confirm our assumptions.

Yet, both SL Nb-MXenes showed the smallest changes, compared to the zero-value. This clearly confirms their fair stability in microalgae growth medium. In addition, we evaluated if the presence of our green microalgae can impact the stability of Nb-MXenes in the culture medium. The results of ζ-potential and conductivity for MXenes after interacting with microalgae in nutritive medium and cultivated over time can be found in the SI (Figs. S9 and S10). Interestingly, we observed that the presence of microalgae seems to stabilize the dispersions of both MXenes. In the case of SL Nb_2_CT_*x*_, the value of ζ-potential even decreased slightly with time towards a more negative value (−15.8 vs.−19.1 mV after 72 h of incubation). The ζ-potential of SL Nb_4_C_3_T_*x*_ increased a little, but after 72 h it still shows much greater stability than the nanoflakes without the presence of microalgae (−18.1 vs. −9.1 mV).

We have also detected lower conductivity of the Nb-MXene solutions incubated in the presence of microalgae, which suggests fewer ions in the nutritive medium. Notably, the instability of MXenes in water is primarily related to surface oxidation^57^. Therefore, we suspected that the green microalgae somehow sweep the generated oxides from the surface of Nb-MXenes, or even prevented their appearance (MXene oxidation). This could be verified by studying the type of matter taken up by microalgae.

### Potential uptake of Nb-MXenes by green microalgae cells

While our ecotoxicological studies showed the ability of microalgae to overcome the toxicity of Nb-MXenes with time and the unusual inhibition-to-stimulation growth manner, we targeted our search to studying the possible mechanisms of action. When organisms like algae find themselves in the presence of compounds or materials unfamiliar to their ecosystem, they may respond with a wide range of activities^58,59^. In the case of nontoxic metal oxides, microalgae may nourish themselves, which results in their constant growth^60^. Upon ingestion of toxic ones, defense mechanisms may be activated, for instance, a change in morphology or shape. The possibility of uptake also needs to be taken under consideration^58,59^. Notably, any signs of defense mechanisms are clear indicator of the toxicity of investigated compound. Therefore, in our further work, we studied potential surface interactions between SL Nb-MXene nanoflakes and microalgae using SEM and the possible uptake of Nb-based MXenes with X-ray fluorescence spectroscopy (XRF). Note that SEM and XRF analyses were performed only in the highest MXene concentration to challenge the activated toxicity issue.

**Figure 4:**
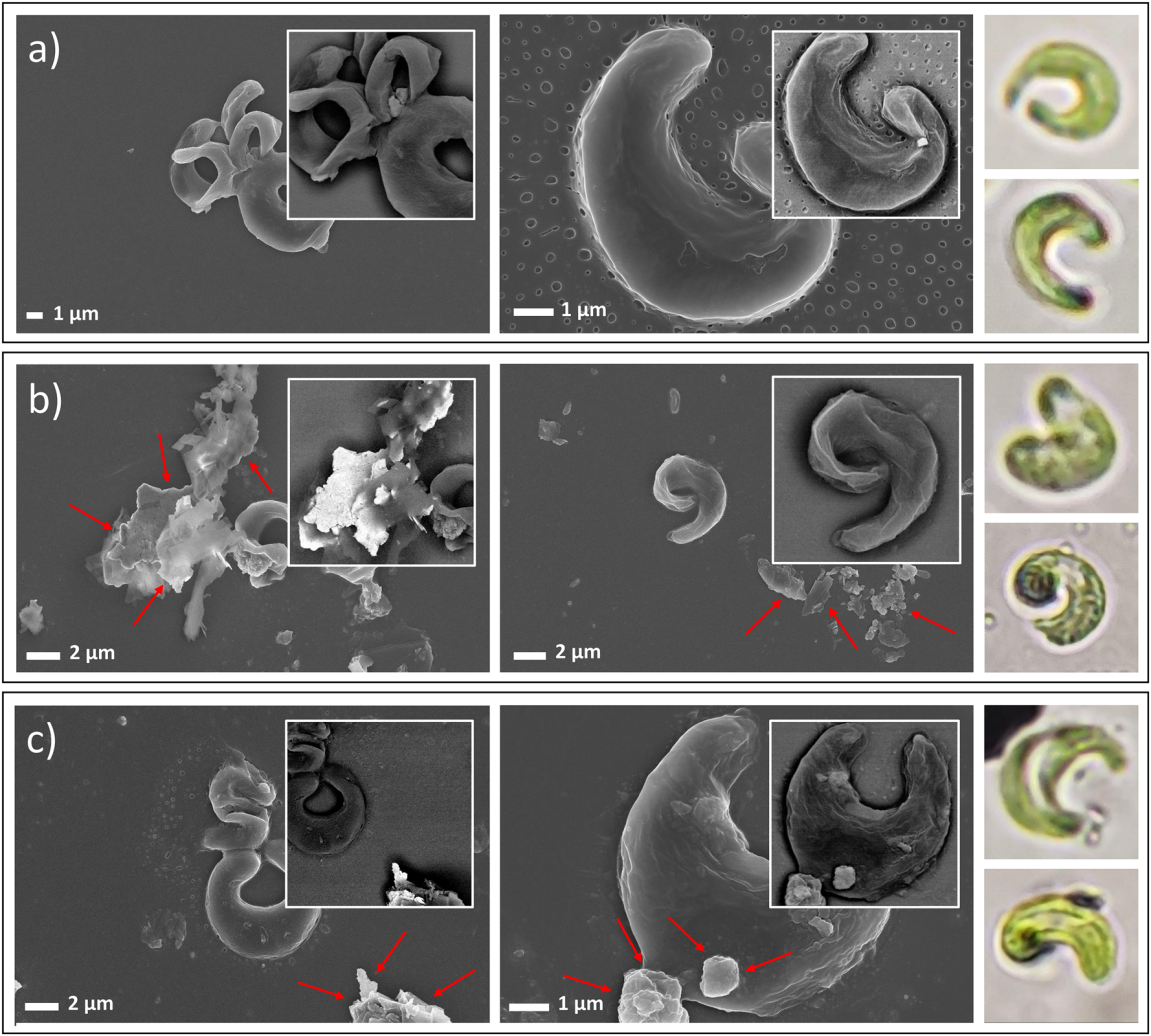
SEM images showing surface interactions between green microalgae and MXene nanosheets after 72 h of interaction with extreme concentration (100 mg L^−1^). (**a**) Untreated green microalgae**,** and after interaction with SL (**b**) Nb_2_CT_*x*_ and (**c**) Nb_4_C_3_T_*x*_ MXenes. Note that Nb-MXene nanoflakes are marked with red arrows. For comparison purposes, photos from a light microscope have been also added.

The SEM results are presented in Fig. 4. The untreated microalgae cells (check Fig. 4a, reference sample) clearly indicate a typical morphology and croissant-like cell shape of *R. subcapitata*. The cells appear to be flattened and a little disturbed. Some microalgae cells are overlapped and wrapped around each other, but this is most likely due to the sample preparation process. Overall, pure microalgal cells had a smooth surface and did not exhibit any morphology changes.

In contrast, microalgae cells with the adsorbed SL Nb-MXene nanoflakes were disturbed (see Fig. 4b, c, red arrows). In the case of Nb_2_CT_*x*_ MXene (Fig. 4b), microalgae tended to grow with the attached 2D nanoflakes, which could plausibly change their morphology. Notably, we observed these changes also under a light microscope (details are presented in SI, Fig. S11). Such morphological transformation has a reasonable basis in microalgae physiology and their ability to protect themselves by changing cell morphology such as cell volume increase^61^. Therefore, it was important to check the number of microalgae cells that were indeed in contact with Nb-MXenes. The SEM investigations have shown that about 52% of microalgal cells were in contact with the Nb-MXenes, while 48% of them avoided the contact. For SL Nb_4_C_3_T_*x*_ MXene, the microalgae attempted to avoid contacting MXene, thus localizing and growing away from the 2D nanoflakes (Fig. 4c). However, we did not observe any nanoflakes penetrating into microalgal cells and therefore causing damage to them.

The self-protection is also a time-dependent response action against hindered photosynthesis, resulting from the adsorption of particles on the cell’s surface and the so-called shading (shadowing) effect^62^. It is evident that every object occurring between microalgae and the light source (i.e., a Nb-MXene nanoflake) will limit the amount of light absorbed by chloroplasts. Yet, we do not suspect it to have a significant impact on the results obtained. As proved by our microscopical observations, even if microalgae cells were in contact with Nb-MXenes, the 2D nanoflakes did not wrap or stick entirely to the surface of the microalgae. Instead, it seems that the nanoflakes were faced edge-on towards microalgae cells and did not cover their surface. Such a set of nanoflakes/microalgae could not significantly limit the amount of light absorbed by the microalgae cells. More importantly, some studies even confirmed the improvement of light absorption by photosynthetic organisms in the presence of 2D nanomaterials^63–66^.

As the SEM images could not directly confirm the uptake of niobium by microalgal cells, our further studies moved to X-ray fluorescence (XRF) and X-ray photoelectron spectroscopy (XPS) analysis to clarify this issue. Thus, we compared Nb-peak intensity for reference microalgae samples that did not interact with MXenes, MXene nanoflakes detached from the surface of microalgae cells, and microalgae cells after removing the attached MXene. Notably, if there is no Nb uptake, the Nb values obtained for microalgae cells should equal zero after removing the attached nanoflakes. Therefore, if Nb uptake occurs, both XRF and XPS results should exhibit clear Nb peaks.

**Figure 5:**
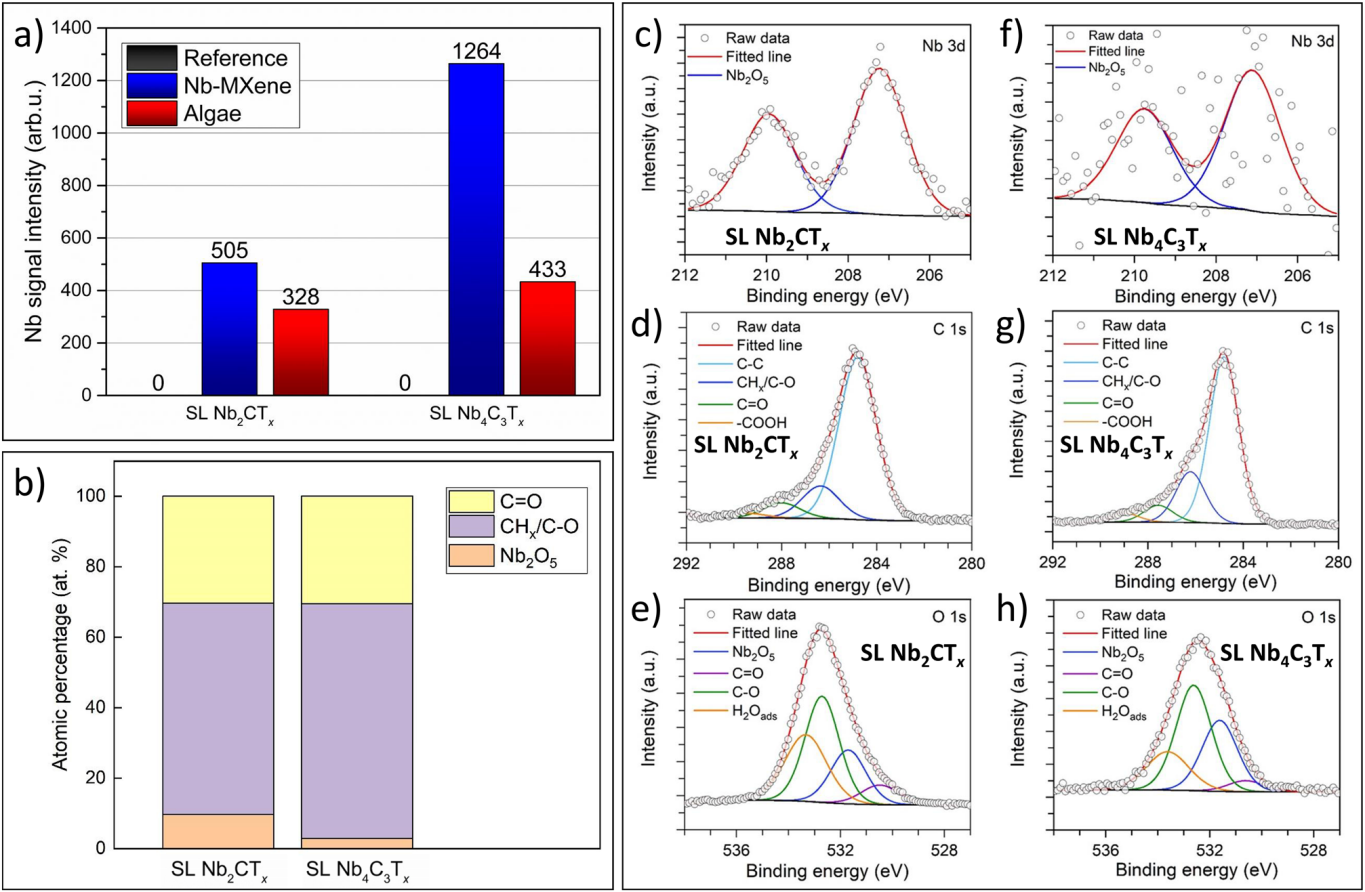
(**a**) XRF results of elementary Nb uptake by green microalgae cells incubated for 72 h in the extreme concentration (100 mg L^−1^) of SL Nb-MXenes. Results show the amount of elementary Nb present in pure microalgae cells (reference sample, grey bars), 2D nanoflakes detached from the surface microalgae cells (blue bars), and microalgae cells after detaching 2D nanoflakes from the surface (red bars), (**b**) chemical composition percentage of microalgae organic components (C=O and CH_x_/C–O) and Nb-oxides present in microalgae cells after incubation with SL Nb-MXenes, Component peak-fitting of XPS spectra for (**c–e**) SL Nb_2_CT_*x*_ and (**f–h**) SL Nb_4_C_3_T_*x*_ MXenes internalized by microalgae cells.

In the case of XRF spectra, the microalgae samples showed a Nb-peak for both SL Nb_2_CT_*x*_ and Nb_4_C_3_T_*x*_ MXenes after interacting with SL Nb-MXenes (see Fig. 5a, also note that results for MAX and ML MXenes are presented in SI, Figs. S12–S17). Interestingly, the intensities of Nb-peaks in both cases were similar (red bars in Fig. 5a). This suggests that the algae could not take up more Nb and a maximum accumulation capacity of Nb has been reached in the cells, even if two-times more Nb_4_C_3_T_*x*_ MXene attached to microalgae cells (blue bars in Fig. 5a). It is noted that the capacity of metal uptake by microalgae depends on the concentration of metal oxides in the surrounding environment^67,68^. Shamshada et al.^67^ have found that the uptake capacity of freshwater algae decreases with a pH value increase. Raize et al.^68^ observed that marine algae possess about 25% higher metal uptake capacities in the case of Pb^2+^ than Ni^2+^.

Therefore, we anticipate that the Nb could be taken up by the algae cells in the form of an oxide. To verify this, we carried out XPS studies for both Nb_2_CT_*x*_ and Nb_4_C_3_T_*x*_ MXenes and algae cells. The results for microalgae after interacting with Nb-MXenes and MXenes separated from the algae cells are presented in Fig. 5b. As expected, we detected the Nb 3d peaks in microalgae samples after removing MXene from their surface. The quantitative amounts of C=O, CH_x_/C-O, and Nb-oxides were calculated based on Nb 3d, O 1s, and C 1s spectra, as obtained from microalgae incubated with SL Nb_2_CT_*x*_ (Fig. 5c–e) and SL Nb_4_C_3_T_*x*_ (Fig. 5f–h) MXenes. Details on peak parameters and global chemical compositions obtained from fitting are displayed in Table S1–3. Notably, the Nb 3d region for SL Nb_2_CT_*x*_ and SL Nb_4_C_3_T_*x*_ (Fig. 5c, f) was fitted by a single Nb_2_O_5_ component. Here, we did not find any MXene-related peaks in the spectra, indicating the microalgae cells only take up the oxide form of Nb. Furthermore, we fitted C 1 s spectra with C–C, CH_x_/C–O, C=O, and –COOH components. We assigned CH_x_/C–O and C=O peaks with the organic contribution of microalgae cells. These organic components comprise the contribution of 36% and 41% C 1s peak in SL Nb_2_CT_*x*_ and SL Nb_4_C_3_T_*x*_, respectively. Next, we fitted O 1s spectra of SL Nb_2_CT_*x*_ and SL Nb_4_C_3_T_*x*_ with Nb_2_O_5_, organic microalgae components (CH_x_/C-O), and surface adsorbed water.

Finally, the XPS results clearly indicate the form of Nb instead of simply its presence. On the basis of the Nb 3d signal position and deconvolution outcome, we confirmed that the Nb was taken up only in the form of an oxide, rather than as ions or as the MXene itself. Moreover, the XPS results have shown a greater capability of microalgae cells to uptake Nb-oxide origination from SL Nb_2_CT_*x*_ than SL Nb_4_C_3_T_*x*_ MXene.

### Tracking the MXene decomposition in presence of green microalgae

While our results on Nb-uptake are exciting and enabled us to recognize MXene decomposition, there was no approach available for tracking the associated morphological changes of 2D nanoflakes. Therefore, we further decided to develop a suitable methodology that could give the direct response to any changes that occurred in both 2D Nb-MXene nanoflakes and microalgae cells. Importantly, we made the case that if the interacting species undergo any transformation, decomposition or defragmentation, this should rapidly appear as a change in shape parameters such as equivalent circular area diameter, circularity, Feret width, or Feret length. As these parameters are suitable for describing elongated grains or 2D nanoflakes, tracking them via dynamic particle shape analysis would give us a valuable insight into morphological transformations of SL Nb-MXene nanoflakes during the remediation process.

**Figure 6:**
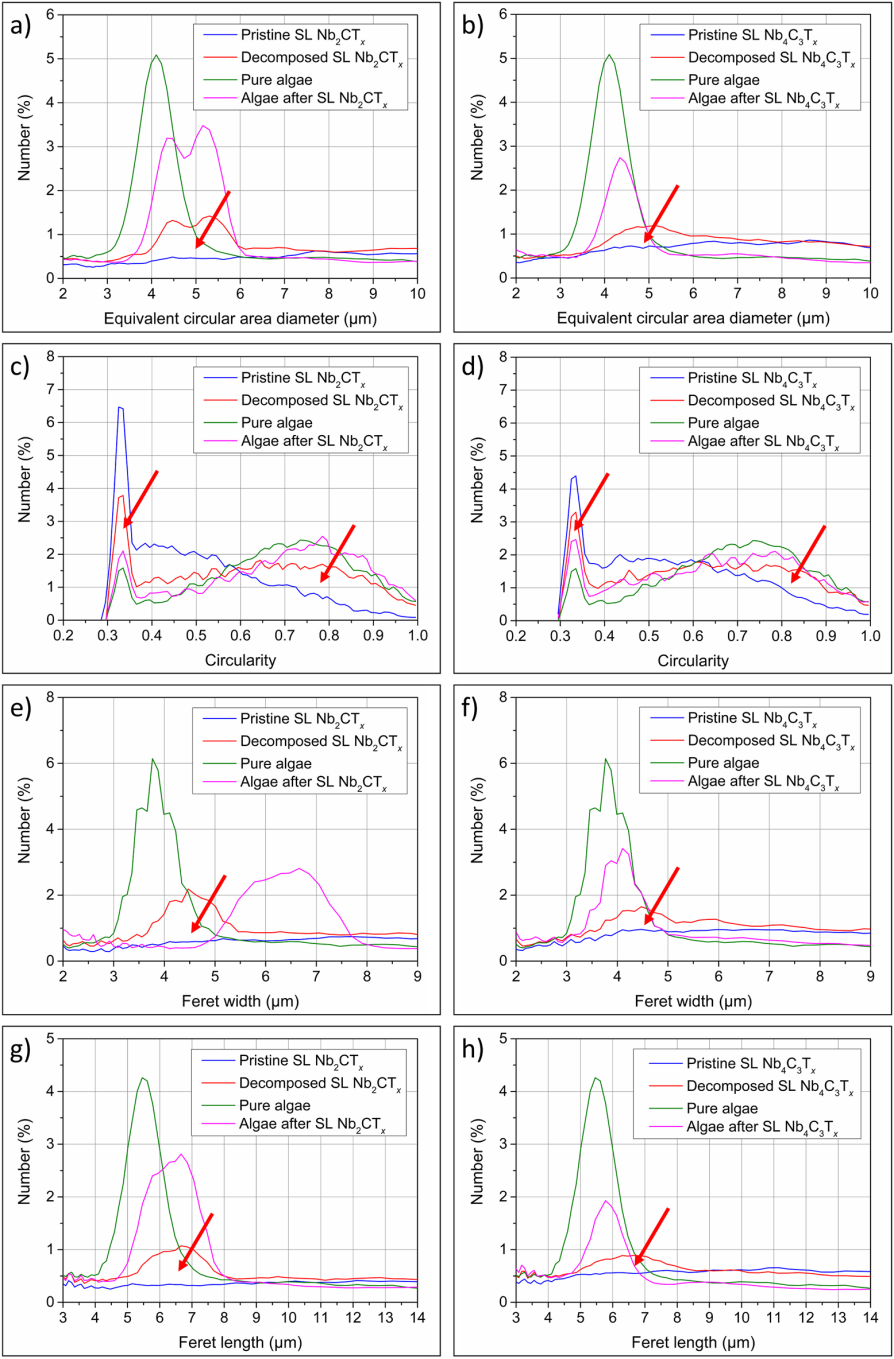
MXene transformation analysis after interacting with green microalgae. The dynamic particle shape analysis considered parameters such as (**a, b**) equivalent circular area diameter, (**c, d**) circularity, (**e, f**) Feret width and (**g, h**) Feret length. For this purpose, two reference microalgae samples were analyzed as well as the pristine SL Nb_2_CT_*x*_ and SL Nb_4_C_3_T_*x*_ MXenes, SL Nb_2_CT_*x*_ and SL Nb_4_C_3_T_*x*_ MXenes decomposed by microalgae and microalgae after treatment with SL Nb_2_CT_*x*_ and SL Nb_4_C_3_T_*x*_ MXenes. The red arrows indicate transformation in investigated shape parameters of 2D nanoflakes.

The obtained results are presented in Fig. 6. For comparison purposes, we additionally tested parental MAX phases and ML MXenes (see SI, Figs. S18 and S19). The dynamic particle shape analysis showed an obvious change in all the shape parameters of both SL Nb-MXenes after interacting with microalgae. As shown by equivalent circular area diameter parameter (Fig. 6a, b), lowering the peak intensity for a fraction of large nanoflakes indicates their tendency to decompose into smaller pieces. Figure 6c,d show a decrease of the peak related to flake lateral size (nanoflake elongation) thus suggesting the transformation of 2D nanoflakes into more particle-like shape. Figure 6e–h, show Feret width and length, respectively. Feret width and length are complementary parameters and should therefore be considered together. After 2D Nb-MXene nanoflakes were incubated in the presence of microalgae, their Feret-related peaks became shifted and decreased their intensity. On the basis of these results combined with morphology, XRF and XPS we conclude that the observed changes were strictly related to the oxidation, as oxidized MXenes become more crumpled and decompose into fragmented and spherical oxide particles^69,70^.

As the shape parameter analysis is highly robust, it may also shed some light on the morphological changes of microalgae cells. We therefore analyzed the equivalent circular area diameter, circularity, and Feret width/length for pure microalgae cells and cells after interacting with 2D Nb-nanoflakes. Figure 6a–h show changes in algae cells’ shape parameters as evidenced by decreased peak intensities as well as maxima shifted to higher values. In particular, the cells’ circularity parameter showed a decrease of elongated cells together with an increase of spherical ones (Fig. 6a, b). Also, the cells’ Feret width increased by several micrometers after interacting with SL Nb_2_CT_*x*_ MXene (Fig. 6e) in contrast to SL Nb_4_C_3_T_*x*_ MXene (Fig. 6f). We suspect that this may be due to the intense uptake of Nb-oxides by microalgae while interacting with SL Nb_2_CT_*x*_. The less severe attachment of Nb-flakes to their surface could cause cells to grow with minimized shading effect.

Our observations on changes in the shape and size parameters of microalgae are complementary to other studies. Green microalgae may alter their morphology to respond to environmental stress by modulating cell size, shape or changing metabolism^61^. For instance, changing the cell size facilitates nutrient uptake^71^. Smaller algae cells exhibit lower nutrient uptake together with disturbed growth rates. Conversely, larger cells tend to consume more nutrients, which are then stored inside the cells^72,73^. Machado and Soares found that cell size may be increased by the biocide triclosan. They also detected profound alteration of algae shape^74^. Also, Yin et al*.*^9^ revealed changes in the morphology of algae after they were in contact with reduced graphene oxide nanocomposites. Therefore, it has become clear that changing size/shape parameters by microalgae is the effect induced by the presence of MXene. Since such changes to size and shape indicate changes in nutrient uptake, we believe that the analysis of the size and shape parameters over time can demonstrate the uptake of niobium oxides by microalgae in the presence of Nb-MXenes.

What is more, MXenes could oxidize in the presence of algae. Dalai et al*.*^75^ noticed uneven morphology of green algae exposed to the nano TiO_2_ and Al_2_O_3_^76^. While our observations were similar to this study, only in the presence of 2D nanoflakes instead of nanoparticles, investigation of the bioremediation effects in terms of the products of MXene decomposition is relevant. As MXenes can decompose into metal oxides^31,32,77,78^, it is plausible that our Nb-nanoflakes could also form Nb-oxides after interacting with microalgae cells.

**Figure 7:**
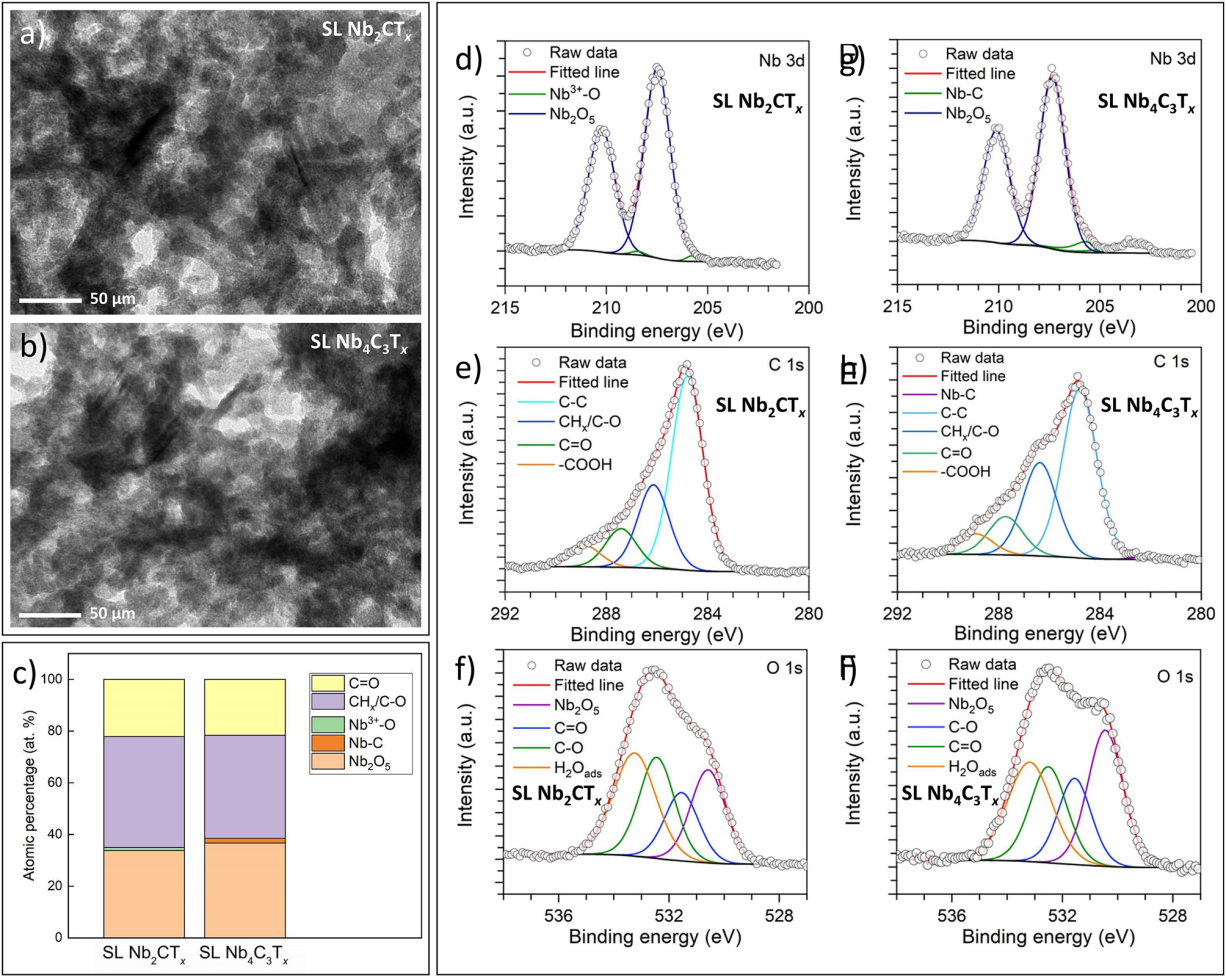
HRTEM images show the morphology of oxidized (**a**) SL Nb_2_CT_*x*_ and (**b**) SL Nb_4_C_3_T_*x*_ MXenes, results of XPS analysis showing (**c**) the composition of oxide products after remediating, component peak-fitting of XPS spectra for (**d–f**) SL Nb_2_CT_*x*_ and (**g–i**) SL Nb_4_C_3_T_*x*_ after remediation with green microalgae.

To explain the 2D-Nb nanoflake remediation via a decomposition mechanism based on oxidation processes, we performed a study using high-resolution transmission electron microscopy (HRTEM) (Fig. 7a, b) and X-ray photoelectron spectroscopy (XPS) (Fig. 7c–i, and Tables S4–5). Both methods are suitable and complementary to studying the oxidation of 2D materials. The HRTEM enables analysis of the degradation of the 2D layered structure and consequent appearance of metal oxide nanoparticles, while XPS is sensitive to surface compounds. For this purpose, we tested 2D Nb-MXene nanoflakes that were extracted from the microalgae cell dispersions, i.e., their form after interacting with microalgae cells (see Fig. 7).

HRTEM studies confirmed the oxidation of both types of Nb-MXene nanoflakes. While the nanoflakes, to some extent, maintain their 2D morphology, oxidation resulted in the appearance of many nanoparticles that covered the surface of the MXene nanoflakes (see Fig. 7a, b). Judging from the results of XPS analysis, c for both the Nb 3d and O 1s signals, show that Nb-oxides have been produced in both cases. As shown in Fig. 7c, the 2D Nb_2_CT_*x*_ and Nb_4_C_3_T_*x*_ MXenes have Nb 3d signals indicating the presence of NbO and Nb_2_O_5_ oxides, while the O 1s signals indicate the amount of O–Nb bonds related to the functionalization of 2D nanoflakes surfaces. We observed that the contribution of Nb-oxide was dominant, compared to Nb–C and Nb^3+^–O.

Figure 7g–i shows the XPS spectra of Nb 3d, C 1s, and O 1s for SL Nb_2_CT_*x*_ (see Fig. 7d–f) and SL Nb_4_C_3_T_*x*_ MXene detached from microalgae cells. The details of peak parameters for Nb-MXenes are given in Tables S4–5, respectively. First, we analyzed the Nb 3d component. Unlike in Nb taken up by microalgae cells, in the MXene detached from microalgae cells, we found other components besides Nb_2_O_5_. In SL Nb_2_CT_*x*_, we observed Nb^3+^-O with a contribution of 15%, while the rest of Nb 3d spectra were dominated by Nb_2_O_5_ (85%). Furthermore, the SL Nb_4_C_3_T_*x*_ sample was fitted with Nb-C (9%) and Nb_2_O_5_ components (91%). Here, the Nb-C comes from the two inner metal carbide atom layers in SL Nb_4_C_3_T_*x*_. Next, we fitted the C 1s spectra with four different components as we did in internalized samples. As expected, the C 1s spectra were dominated by graphitic carbon, followed by the contribution from organic species coming from microalgae cells (CH_x_/C–O and C=O). Moreover, in the O 1s spectra, we observed a contribution from organic species of microalgae cells, Nb-oxide, and adsorbed water.

Further, we checked whenever the decomposition of Nb-MXenes is connected with the presence of reactive oxygen species (ROS) in the nutritive medium and/or in the microalgae cells. For this purpose, we evaluated singlet oxygen (^1^O_2_) levels in medium and intracellular glutathione—a thiol that acts as microalgae antioxidant. The results are presented in the SI (Figures S20 and S21). The cultures with SL Nb_2_CT_*x*_ and Nb_4_C_3_T_*x*_ MXenes were characterized by decreasing amounts of ^1^O_2_ (see Fig. S20). In the case of SL Nb_2_CT_*x*_ MXene, the ^1^O_2_ decreased to about 83%. For the microalgae culture with SL Nb_4_C_3_T_*x*_, the ^1^O_2_ dropped even more, up to 73%. Interestingly, the ^1^O_2_ changes showed the same trend as the previously observed inhibition-to-stimulation effect (check Fig. 3). One could argue that incubation with extensive light could potentially alter photooxidation. Nevertheless, the results from the control assay showed an almost constant level of ^1^O_2_ for the duration of the experiment (Fig. S22). We also observed the same decreasing trend in the case of intracellular ROS levels (see Fig. S21). Initially, the ROS levels in the microalgae cells incubated in the presence of SL Nb_2_CT_*x*_ and Nb_4_C_3_T_*x*_ exceeded the levels detected for pure microalgae cultures. However, in the end, microalgae seemed to adapt to the presence of both Nb-MXenes, as the ROS levels decreased to 85 and 91% of the levels measured for pure microalgae culture inoculated with SL Nb_2_CT_*x*_ and Nb_4_C_3_T_*x*_, respectively. This may suggest that with time, microalgae felt more comfortable in the presence of Nb-MXene than in a nutritive medium alone.

Microalgae are a diverse group of photosynthetic organisms. During photosynthesis, they convert atmospheric carbon dioxide (CO_2_) to organic carbon. The products of photosynthesis are glucose and oxygen^79^. We suspect that oxygen generated in such a way plays a vital role in the oxidation of Nb-MXenes. One of the possible explanations for that is an aeration differential parameter being formed at low and high oxygen partial pressures, outside and within an Nb-MXene nanoflake. It means that whenever there are areas with different oxygen partial pressures, the areas where the level is lowest, forms the anode^80–82^. Here, microalgae can contribute to establishing differential aeration cells on the surface of MXene flakes, as they produce oxygen due to their photosynthetic nature. As a result, biocorrosion products (here Nb-oxides) are being formed. Another aspect is that microalgae can produce organic acids, which are excreted into the water^83,84^. Consequently, a corrosive environment is formed, which transforms the Nb-MXenes. Also, microalgae can change the environment's pH to alkaline due to CO_2_ uptake, which can also be corrosive^79^.

What is more, a darkness/light photoperiod applied in our study is crucial in understanding the obtained results. This aspect was described in detail in the work of Djemai-Zoghlache et al*.*^85^ They intentionally applied a photoperiod of 12/12 h to showcase the biocorrosion involved in biofouling with red microalgae *Porphyridium purpureum*. They showed that the photoperiod relates to the evolution of the free potential of biocorrosion, which appears as pseudo-cyclical swings of approximately 24:00. Such observations were confirmed by Dowling et al.^86^ They proved that photosynthetic biofilms of cyanophyceae *Anabaena sp.* produce dissolved oxygen under the influence of light, which is involved with variations or oscillations of the free potential of biocorrosion. The importance of photoperiod underlines the fact that the free potential of biocorrosion increases in the light phase and decreases in the dark one. It's connected with the oxygen the photosynthetic microalgae produce, which influences the cathodic reaction by the partial pressures created near the electrode^87^.

Fourier-transform infrared spectroscopy (FTIR) was further performed to check if any changes occurred in the chemical composition of microalgae cells after interacting with Nb-MXenes. These obtained results were complex, and we have presented them in the SI (Figs. S23–S25, including results from MAX phases and ML MXenes). Briefly, the obtained spectra for reference microalgae gave us important information about the chemical characteristics of these organisms. These are most likely vibrations located at 1060 cm^−1^ (C–O), 1540 cm^−1^, 1640 cm^−1^ (C=C), 1730 cm^−1^ (C=O), 2850 cm^−1^, 2920 cm^−1^ (C–H) and 3280 cm^−1^ (O–H). For SL Nb-MXenes, we detected stretching vibrations characteristic of C–H bonds, which correspond with our previous study^38^. However, we observed some additional drops of peaks associated with C=C and C–H bonds. This suggests a possibility of minor changes in microalgae chemical composition due to interacting with SL Nb-MXenes.

While considering the possible changes in the biochemistry of microalgae, it is essential to revisit the accumulation of inorganic oxides (such as Nb-oxides)^59^. It is related to the absorption of metal on the cell surface, transportation into the cytoplasm, bonding with intracellular carboxyl groups, and accumulation in microalgae polyphosphate bodies^20,88–90^. Moreover, bonding between microalgae and metals is supported by functional groups exhibited by the cell. For this reason, uptake also depends on the surface chemistry of microalgae, which is rather complicated^9,91^. Overall, as expected, the chemical composition of green microalgae slightly changed because of Nb-oxide uptake.

Interestingly, the observed initial inhibition of microalgae was reversible as time passed. As we observed, microalgae overcame the initial change in their surrounding environment to finally return to their normal growth rate or even increasing it. The ζ-potential studies showed a high stability when introduced into the nourishing medium. Therefore, surface interactions between microalgae cells and Nb-MXene nanoflakes were maintained during the whole remediation experiment. In our further analysis, we concluded the potential mechanisms of action behind this extraordinary microalga behavior.

SEM observations showed the tendency of microalgae to attach to Nb-MXenes. With the dynamic image analysis technique, we confirmed that this effect resulted in the transformation of 2D Nb-MXene nanoflakes into more spherical particles, thus proving the decomposition of the nanoflakes, which we linked to their oxidation. To verify our hypothesis, we performed a set of material and biochemical studies. As detected, nanoflakes were gradually oxidized and decomposed into NbO and Nb_2_O_5_ products, which do not seem to pose a threat to green microalgae. With FTIR observations, we did not detect significant changes in chemical compositions of microalgae incubated in the presence of 2D Nb-MXene nanoflakes. Keeping in mind the possibility of microalgae uptake of Nb-oxides, we performed XRF analysis. These results clearly show that investigated microalgae nourished themselves with niobium oxides (NbO and Nb_2_O_5_), which were nontoxic towards studied microalgae.

Microalgae cell transformations were further confirmed by dynamic image analysis. As shown by Feret width and length parameters, microalgae tended to develop wider and shorter cells. Moreover, the average diameter of microalgal cells increased by about 1–2 μm, which supports our swelling observation.

As decomposition of Nb-MAX and Nb-MXenes by microalgae is possible, we suspect different mechanisms of action to be responsible for the time-dependent decrease of microalgal growth, which is surface physicochemical interactions between MXene and microalgae cells. After getting into close contact, the MXene-microalgae interface is created. Next, the 2D nanosheets oxidize and decompose into Nb-oxides, which are then taken up by the cells. Interestingly, such a mechanism helps in both reducing toxicity and in returning microalga to their normal growth.

## Conclusion and outlook

In summary, we successfully obtained single-layered 2D nanoflakes of Nb_2_CT_*x*_ and Nb_4_C_3_T_*x*_ MXenes using HF-etching and delamination with TBAOH. The synthesized Nb-MXenes were further characterized with SEM and HRTEM. With those techniques, we were able to confirm their layered structure and the synthesis efficiency. An essential aspect of applying every newly designed nanostructure is the ability to remove it from the environment when necessary. Therefore, we have studied the bioremediation of Nb-based MXenes by the microalgae *R. subcapitata*. These organisms are non-demanding producers of the freshwater food web, and can be used as effective toxicity indicators. Our studies have shown no acute or chronic toxicity of SL Nb-MXenes towards the investigated microalgae. Only a slight reduction of microalgal growth was detected for SL Nb-MXene nanoflakes in their extreme concentrations (100 mg L^−1^).

The bioremediation by microalgae consists of the decomposition of Nb-based MXenes due to physicochemical interactions between MXene and microalgae cells. At a first glance Nb-MXene nanoflakes attached to microalgae surface, which slightly reduced their growth. With prolonged time, microalgae remediated the Nb-MXenes by oxidizing them and further decomposing them into nontoxic NbO and Nb_2_O_5_ oxides. This step was associated with algal uptake of the oxides, which enabled further recovery after 72 h of water treatment. Such effects further influenced the shape parameters of both microalgae cells and 2D Nb-MXene nanoflakes due to nutritional and decomposition processes, respectively.

Our results clearly show the potential for Nb-based MXenes application in different fields due to their lack of acute or chronic toxicity. Slight growth inhibition occurred only in conditions similar to unusual circumstances, such as an accidental release to the environment. Even so, microalgae were able to survive in such an unfriendly environment, and return to their normal or even increase growth in a relatively short time (72 h). Our study increases the understanding of material surface-organism interactions. The mechanism of action showing reduced ecotoxicity of Nb-based 2D nanoflakes in connection with the bioremediation process lays the groundwork towards ecological and environmental management of 2D nanomaterials. In addition, our results provide foundations for further studies on niobium mining with a fully green approach. However, we must stress that we obtained our results in a fully controlled model environment, so comprehensive research performed in a real-world environment is needed.

In addition, by using 2D MXene nanomaterials as a model system, we have developed a facile approach for tracking even fine materials shape transformation. This approach was based on dynamical shape parameters analysis specially designed for tracking the bioremediation process. By testing the equivalent circular area diameter, circularity, Feret width and Feret length, it was possible to observe the decomposition of 2D flakes into spherical oxide particles. This method gives evidence and a fundamental basis for investigating various environmental effects caused by the presence of inorganic crystalline nanomaterials. By using a similar approach, the extended short- and long-time tests of the possible 2D materials’ effects in freshwater ecosystems could be further analyzed via simple and controllable manner.

## Methods

### Preparation of niobium-based MAX phases and MXenes

The parent Nb-MAX phases were obtained using direct mixing of precursor powders, which were then pressed and heated. Niobium and aluminum (− 325 mesh) and graphite carbon (APS 7–11 micron) powders were mixed in ratios of 2:1.3:1 and 4:1.5:2.7 (respectively for Nb_2_AlC and Nb_4_AlC_3_) in a Turbula T2F mixer for 3 h at 56 RPM, with 10 mm yttria-stabilized zirconia balls as mixing media. All powders were obtained from Alfa Aesar (Haverhill, MA, USA). The Nb_4_AlC_3_ mixed powders were additionally pressed into 10 g pellets. Obtained samples were then heated in a tube furnace under flowing argon (1600 °C for 4 h and 1700 °C for 1 h, for Nb_2_AlC and Nb_4_AlC_3_ respectively, at a heating rate of 10 °C/min). The obtained samples were cooled down to room temperature and then ground down to -325 mesh.

The multilayer (ML) Nb-MXenes were obtained via selective etching. The MAX phase powders were soaked in 48% hydrofluoric acid (HF) at 40 °C (1 g of MAX per 10 mL of solution) and then stirred for 72 h. Etched powders were washed few times with DI water and centrifuged (3500 RPM, 2401 RCF, 5 min). After reaching a pH of about 6, the samples were dried under vacuum.

The delamination process into single-layer (SL) Nb-MXenes was carried out in an aqueous solution of 50 *wt*% tetrabutylammonium hydroxide (TBAOH, (C_4_H_9_)_4_NOH, Sigma-Aldrich, Darmstadt, Germany) for 24 h at 30 °C. The proportions were 0.5 g of Nb-MXene per 2.5 mL of solution. Then, the mixture of MXene and TBAOH was separated using centrifugation (3500 RPM, 2401 RCF), after which obtained sediment was soaked in 50 mL of degassed double distilled water (DDW). The resulted suspension was then shaken and re-dispersed using ultrasound for 2 h, which also helped in separating nanoflakes from each other. Then, the mixture was centrifuged (3500 RPM, 2401 RCF, 1 h), and the black colloidal solutions of both Nb-MXenes were collected while the sediments were discarded. Colloidal solutions were then washed with DDW multiple times with centrifugation until pH reached about 7. Such prepared solutions were stored at 5 °C for further use.

### Studies on morphology and structure

The morphology of niobium carbide MAX phases, ML, and SL MXenes was investigated using a scanning electron microscopy (SEM). The samples were deposited onto sticky carbon tape and then coated with a layer of carbon using a BAL-TEC SCD 005 sputter coater. Such prepared samples were analyzed with a Zeiss Ultra Plus (Zeiss, San Diego, CA, USA) microscope at an accelerating voltage of 2.0 kV.

The high-resolution transmission electron microscopy (HRTEM, Philips CM 20, Amsterdam, Holland) was performed for the SL Nb_2_CT_*x*_ and Nb_4_C_3_T_*x*_ MXene nanoflakes after delamination in TBAOH and the oxidation process. The samples were placed on Cu-C mesh for HRTEM examination.

X-ray diffraction (XRD, D8 ADVANCE, Bruker, Billerica, MA, USA) was utilized to examine the phase composition of obtained Nb-MXenes. Investigations were carried out using CuK_α_ radiation at a wavelength λ = 0.154056 nm, with a voltage of 40 kV, current of 40 mA, in an angular range of 2–80 °, and with 0.025 ° step.

### Analysis of ecotoxicity towards green microalgae

The ecotoxicity of Nb_2_CT_*x*_ and Nb_4_C_3_T_*x*_ MXenes and Nb_2_AlC and Nb_4_AlC_3_ MAX phases was evaluated using a standardized ALGALTOXKIT F set (MicroBioTests, Gent, Belgium), allowing the estimation of 72 h of microalgal growth inhibition according to OECD Guideline 201^92^ and ISO Standard 8692:2012^93^. The ALGALTOXKIT also permits a preliminary assessment at an early stage of microalgal growth, as the inhibition was also determined after 24 and 48 h of the experiment. The test is based on the measurement of optical density of the microalgae cultures, which can be further easily converted to cell density. The test was carried out using green microalgae *Raphidocelis subcapitata* and microalgal culturing medium analogous to that recommended by the OECD Guideline 201 and ISO Guideline 8692:2012. The culture medium prepared from four stock solutions, with a composition (per liter) as follows: (1) 1.5 g NH_4_Cl, 1.2 g MgCl_2_⋅6H_2_O, 1.8 g CaCl_2_⋅2H_2_O, 1.5 MgSO_4_⋅7H_2_O, 0.16 g KH_2_PO_4_; (2) 64 mg FeCl_3_⋅6H_2_O, 100 mg Na_2_EDTA⋅2H_2_O; (3) 185 mg H_3_BO_3_, 415 mg MnCl_2_⋅4H_2_O, 3 mg ZnCl_2_, 1.5 mg CoCl_2_⋅6H_2_O, 0.01 mg CuCl_2_⋅2H_2_O, 7 mg Na_2_MoO_4_⋅2H_2_O; (4) 50 g NaHCO_3_. The detailed mass concentrations of nutrients are presented in the Supplementary Information (Table S6). To prepare the culture medium, 800 mL of distilled (DI) water was transferred to an Erlenmeyer flask. Next, we added 10 mL of stock solution (1) and 1 mL of each stock solution (2), (3) and (4). Then, we brought the volume to 1000 mL with DI water. After vigorous shaking, we adjusted the pH with 1 M HCl or 1 M NaOH to 8.1 ± 0.2 in the last step.

To carry out our experiment, 0.9 mL of concentrated microalgal inoculum (10^6^ cells mL^−1^, determined by OD_670_ measurement) were transferred to each flask, containing 90 mL of every dilution of the compound. Thus, the initial concentration of microalgae in each flask was 10^4^ cells mL^−1^. Such sufficiently low initial concentration eliminates the risk of nutrient depletion. Potentially toxic concentrations varied from 0.01 mg L^−1^ to 100 mg L^−1^, with the individual dilution factor (IDF) q = 10. Then, 25 mL of the microalgae-toxicant dilutions from each flask were transferred into test vials and incubated at 23 ± 2 °C with sideways illumination of 10,000 lx for three days, under a photoperiod of 24 h of light for 0 h of darkness (24/0; L/D). The microalgal culture without noncompound was used as a negative control sample. After every 24 h, OD_670_ was measured, and the specific growth rates (*µ*) for each test and control batch replicate were calculated from the Eq. (1):

1 $$\mu =\frac{\mathit{ln}{n}_{L}-\mathit{ln}{n}_{0}}{{t}_{L}-{t}_{0}}$$

where *t*_*0*_ is the time of test start, *t*_*L*_ is the time of measurement, *n*_*0*_ is the initial cell density, *n*_*L*_ is the cell density measured at time *t*_*L*_.

In our work, we defined the specific growth rates as biomass doubling rates to show even better the differences which occur over time. The percentage inhibition of microalgal growth (*I*_*µi*_) was calculated based on the determined specific growth rates from Eq. (2):

2 $${I}_{\mu i} (\%)=\frac{{\mu }_{c}-{\mu }_{i}}{{\mu }_{c}}\times 100$$

where *µ*_*i*_ is the growth rate for test batch, *µ*_*c*_ is the mean growth rate for the control batches. The significance of results was estimated using the Student *t*-test with a confidence level of 95%. During the assays, microalgae cultures were resuspended for a few minutes every day to avoid algae settlement and subsequent impact of shadowing effects limiting their growth rate.

### Studies on microalgae mechanisms of action and potential intake

Due to the observed ecotoxicological effect of Nb-MXenes presence, the intake analyses have been performed only in case of *extreme concentrations* (100 mg L^−1^). Four sets of investigated samples were prepared for the tests: pristine nanoflakes, decomposed nanoflakes, pure microalgae and microalgae after treatment with nanoflakes. Microalgae and nanoflakes were separated from each other using the gradient centrifugation method (850 RPM, 142 RCF, 120 s) and subsequent washing with a growing medium. The centrifugation parameters were determined experimentally. Microalgae incubated without MXeneanoflakes were used as a negative control.

In the case of interactions that may occur between Nb-MXene nanoflakes and microalgae, as well as for cells morphology changes investigations, a scanning electron microscope (SEM) accompanied by an energy-selective backscattering (BSE) detector was applied. Cultures of microalgae with nanoflakes were deposited onto the surface of muscovite mica rings (V1, Science Service) which can be utilized for SEM observations. Then, 2.5% (*v/v*) glutaraldehyde (Sigma-Aldrich, Saint Louis, MO, USA) was added. Such prepared samples were left overnight, which allowed cultures to sediment and then they were dried by freeze-drying. After that, mica rings were transferred onto sticky carbon tape and coated with gold. Complementary observations were carried out using the OPTA-TECH MB 200 light microscope (OPTA-TECH, Warsaw, Poland).

The studies on microalgae cells shape were performed using Particle Insight dynamic image analyzer Sentinel Pro (Micromeritics Instrument Corporation, Norcross, GA, USA) equipped with a stroboscope camera and peristaltic pump. The shape parameters were analyzed using an equivalent circular area diameter model (ECAD) in terms of diameter and circularity and an irregular model for Feret width and length.

The presence of niobium in microalgae cells was measured using a PI 100 benchtop X-ray fluorescence spectrometer (XRF, Polon-Izot, Warsaw, Poland). The spectrometer was provided with a silicon drift detector (SSD) of 125–140 eV resolution, a test tube with rhodium (Rh) anode, as well as a multilayer monochromator of 50 keV. Investigations were done for wet samples and in an air atmosphere using a measurement time of 300 s per sample.

The quantitative composition of the chemical state of elements present in microalgae was investigated with X-ray photoelectron spectroscopy (XPS), thus it can also show possible intake of niobium. For this purpose, the PHI 5000 VersaProbe spectrometer (Physical Electronics, Methuen, MA, USA) was utilized, and the absorption/reflectance spectra for a dry mass were studied. Monochromatic X-rays with energy *hν* = 1486.6 eV (AlK_α_) with a power of 23 W were used as the excitation source. Survey and high-resolution XPS spectra were recorded with the energy matching function: 117.4 and 23.5 eV, respectively. The measured bond energies for individual elements detected on the surface of the tested samples were corrected concerning the carbon peak C1s = 284.8 eV.

The levels of intracellular ROS and singlet oxygen were evaluated with fluorescent indicators. To check intracellular ROS, we dissolved CM-H_2_DCFDA (Thermo Fisher Scientific, Waltham, MA, USA) in dimethyl sulfoxide (DMSO, 99.99% pure, Chempur, Piekary Śląskie, Poland) to get a concentration of 0.5 mM. Next, we transferred 230 µL of test culture to the multiwell plate at each experimental stage and added the fluorescent indicator to get the final CM-H_2_DCFDA concentration of 2 µM in each well. The singlet oxygen levels were studied with Singlet Oxygen Sensor Green fluorescent reagent (Thermo Fisher Scientific, Waltham, MA, USA). For this purpose, we dissolved as received reagent in methanol (99.8% pure, WARCHEM, Warsaw, Poland) to obtain the final concentration of 1.4 mM. Further, we transferred 250 µL of each sample into the multiwell plate at each stage of the ecotoxicological test. Next, we added a dissolved fluorescent probe to each well to receive its final concentration of 5.5 µM. In the subsequent step we incubated such prepared samples in the dark for 60 min and measured the fluorescence intensity with a microplate reader (Infinite 200 PRO, Tecan, Männedorf, Switzerland) with the excitation and emission of 485 and 520 nm, respectively. We calculated levels of intracellular ROS and singlet oxygen from Eq. (3):

3 $$N \left(\%\right)=\frac{A}{B}\times 100$$

where A is the fluorescent intensity of the investigated sample, B is the is the fluorescent intensity of the reference sample.

The stability of the Nb-MXene nanoflakes and MAX phases in a culture medium was investigated by ζ-potential and conductivity measurements. For this purpose, a NANO ZS ZEN3500 analyzer (Malvern Instruments, Malvern, UK) equipped with a back-scattered light detector and operating at a 173 ° angle was used. Samples were prepared in a culture medium with the final concentration of Nb-MXenes and MAX phases measuring 100 mg L^−1^ (*extreme concentrations*). Each of the investigated samples was homogenized for 60 s using mild sonication and was studied at 25 °C. Additional investigations of dispersion stability of SL Nb_2_CT_*x*_ and Nb_4_C_3_T_*x*_ MXenes were performed for four sets of investigated samples: pristine nanoflakes, decomposed nanoflakes, pure microalgae and microalgae after treatment with nanoflakes. The separation of microalgae and nanoflakes was performed as described above. Shortly before the measurement, samples were transferred into the capillary cell. We made sure to remove any gas bubbles which could disturb the measurement. We did not employ any additional steps to prepare samples. The measurements were performed after 0, 24, 48, and 72 h, for the samples incubated under conditions analogous to microalgae cultivation. The results were presented as the mean of three subsequent measurements.

Fourier transform infrared spectroscopy for identifying organic, polymeric and inorganic components in microalgae cells (FTIR, Nicolet iS5 FTIR Spectrometer, Thermo Scientific, Waltham, MA, USA) was applied. FTIR analysis has been performed for dry matter obtained by freeze-drying (Alpha 2-4 LD Plus, Martin Christ, Osterode am Harz, Germany).

## Supplementary Information

Supplementary Information.

## Data Availability

All relevant data supporting the key findings of this study are available within the article and its Supplementary Information file or from the corresponding author upon reasonable request.
