# Supplementary material for: Understanding the mechanism of Nb-MXene bioremediation with green microalgae
Source: Sci Rep. 2022 Aug 23;12:14366. doi: 10.1038/s41598-022-18154-3 (PMC9399251; doi:10.1038/s41598-022-18154-3)
Supplement: Supplementary file 1 — Supplementary Information. [file 41598_2022_18154_MOESM1_ESM.docx]

***Supplementary Information***

***Understanding the Mechanism of Nb-MXene Bioremediation with Green Microalgae***

Michał Jakubczak ^1,*^, Dominika Bury ^1^, Muhammad Abiyyu Kenichi Purbayanto ^1^, Anna Wójcik ^2^, Dorota Moszczyńska ^1^, Kaitlyn Prenger ^3^, Michael Naguib ^3^ and Agnieszka Maria Jastrzębska ^1,**^

^1^ Warsaw University of Technology, Faculty of Materials Science and Engineering, Wołoska 141, Warsaw 02-507, Poland

*E-mail: michal.jakubczak.dokt@pw.edu.pl

**E-mail: agnieszka.jastrzebska@pw.edu.pl

^2^ Polish Academy of Sciences, Institute of Metallurgy and Materials Science, W. Reymonta 25, 30-059 Cracow, Poland

^3^ Tulane University, Department of Physics and Engineering Physics, New Orleans, LA 70118, USA


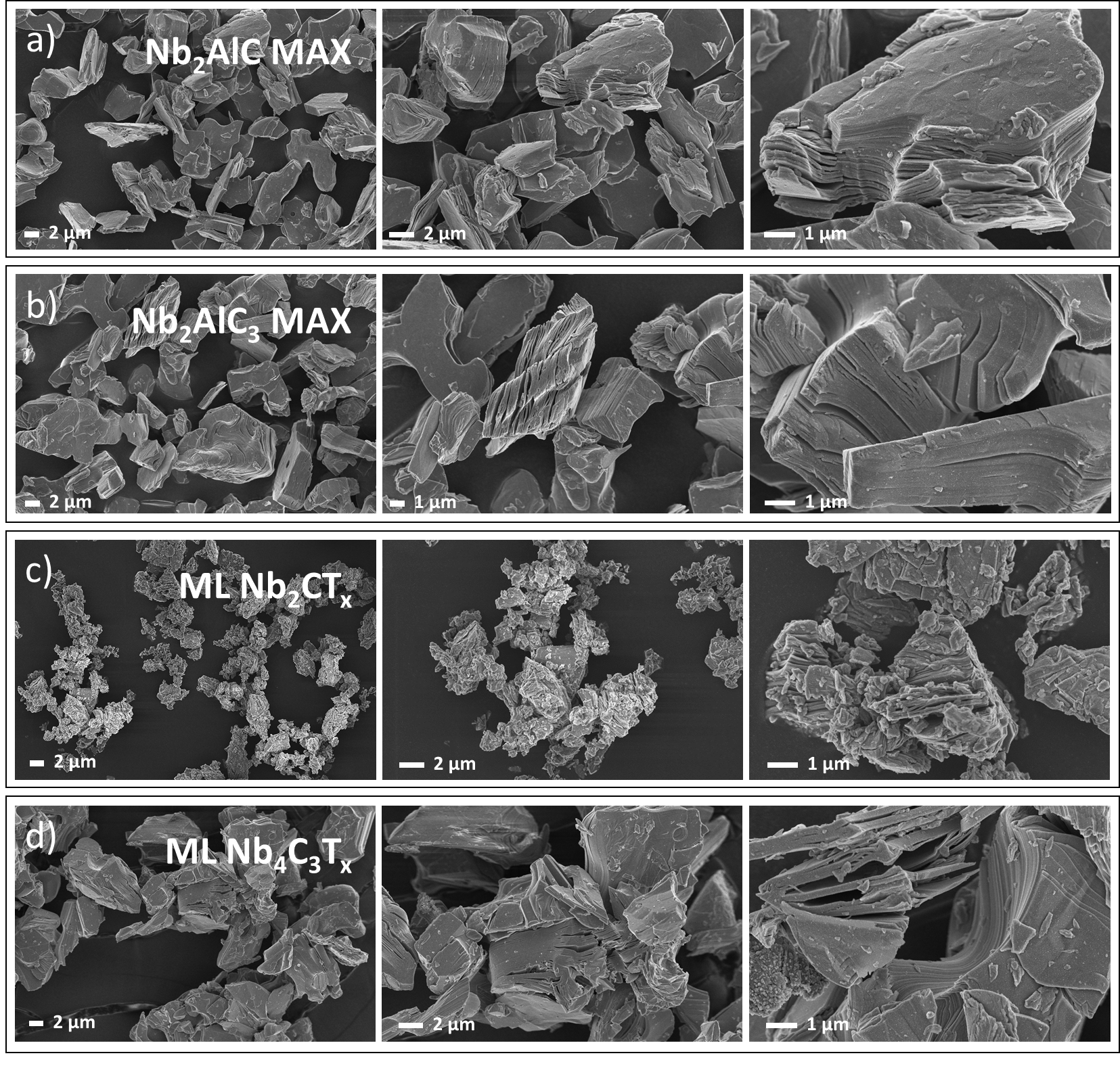


**Figure S1.** SEM images of: **a)** Nb_2_AlC and **b)** Nb_4_AlC_3_ MAX phases, as well as 2D multi-layer: **c)** Nb_2_CT*_x_* and **d)** Nb_4_C_3_T*_x_* MXenes.


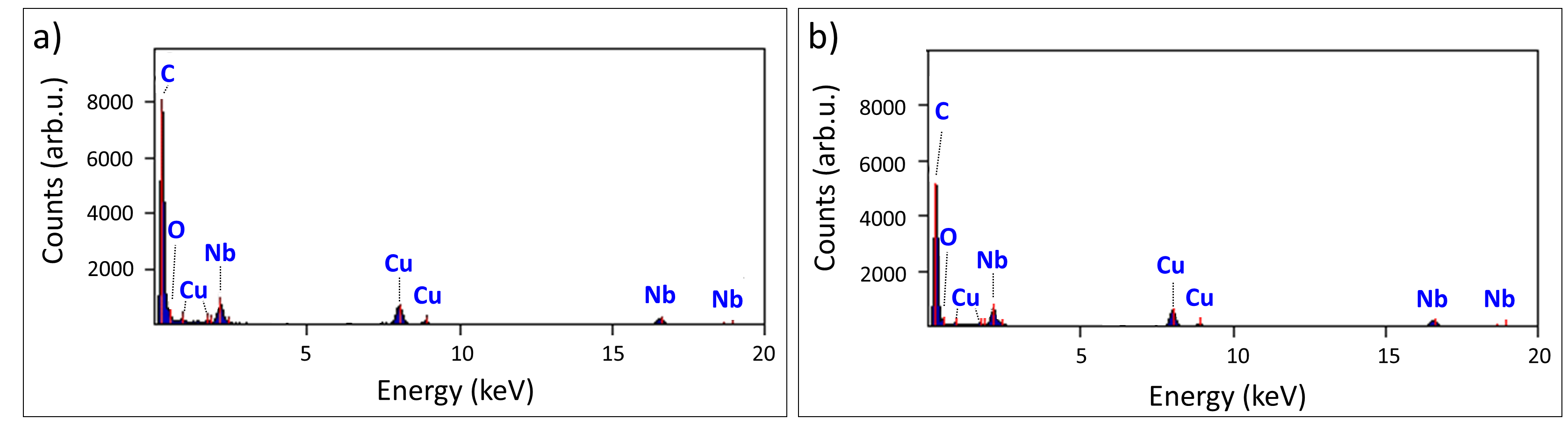


**Figure S2.** Energy dispersive X-ray analysis (EDX) obtained for 2D SL **a)** Nb_2_CT*_x_* and **b)** Nb_4_C_3_T*_x_* MXenes.


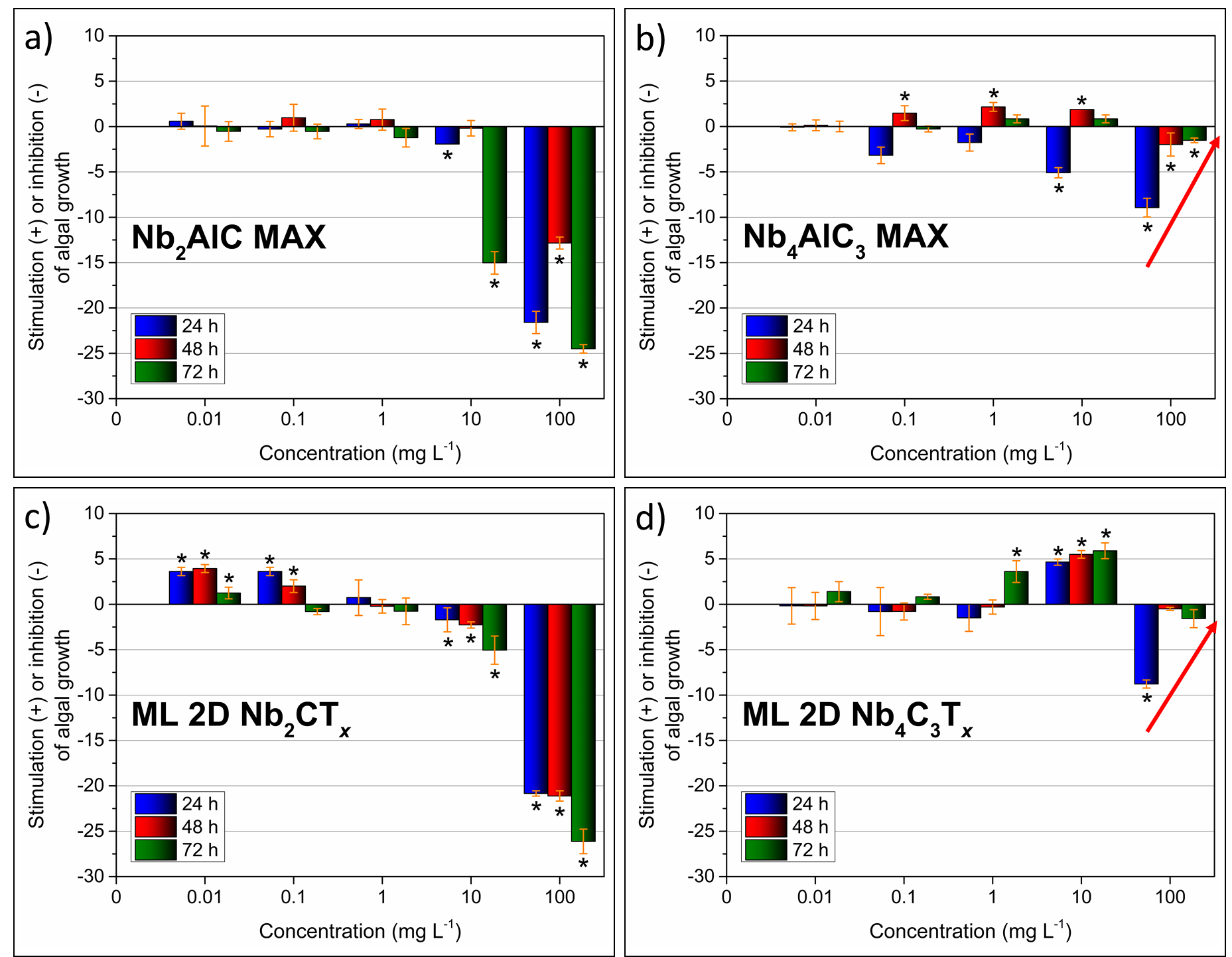


**Figure S3.** Stimulation (+) or inhibition (-) of microalgal growth in the presence of **a)** Nb_2_AlC and **b)** Nb_4_AlC_3_ MAX phases and ML **c)** Nb_2_CT*_x_* and **d)** Nb_4_C_3_T*_x_* MXenes. Significant data (t-test p<0.05) were marked with an asterisk (*). The red arrows indicate the reversal inhibition-to-stimulation effect.


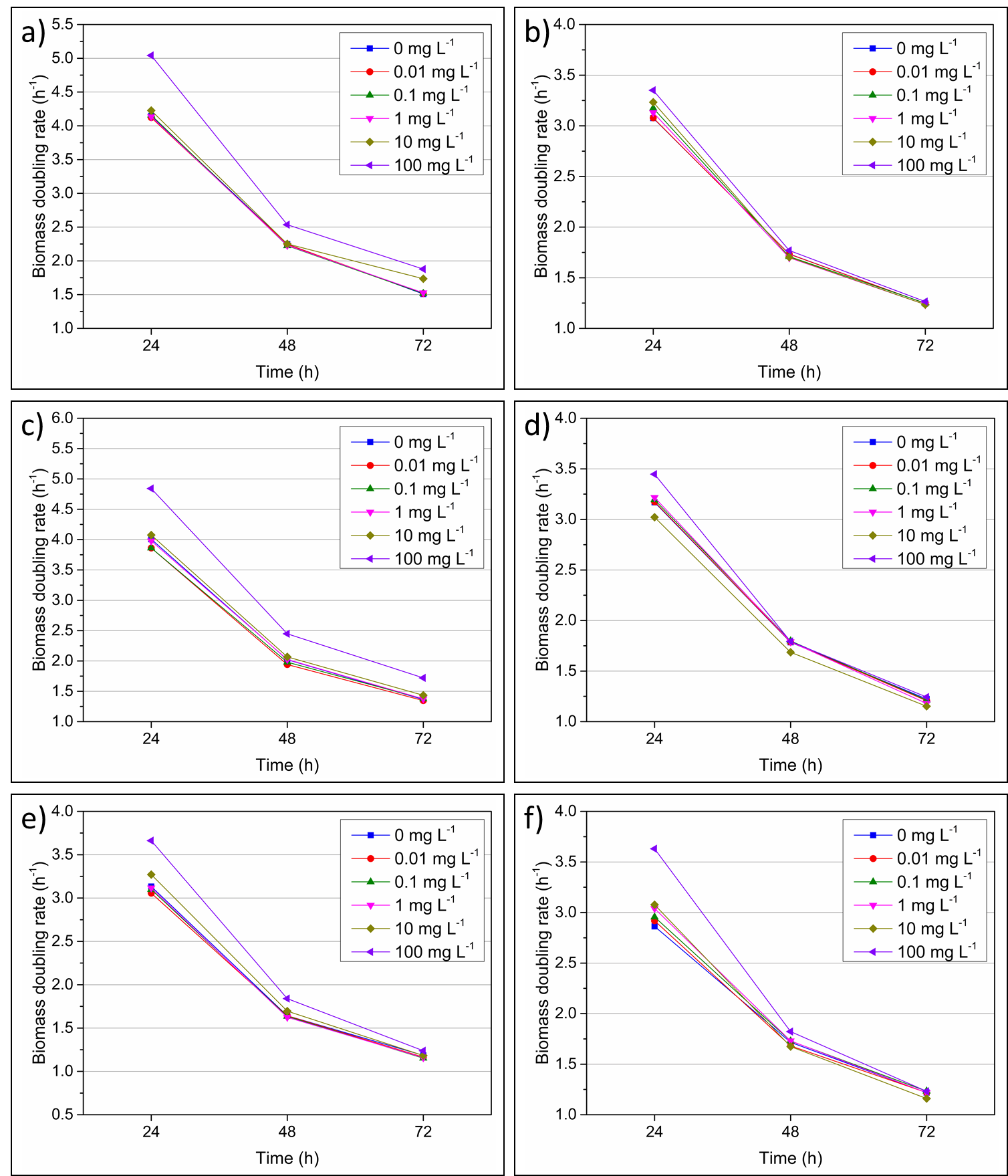


**Figure S4.** Microalgae biomass doubling rate alterations over time evaluated for the test cultures in the presence of: **a)** Nb_2_AlC MAX, **b)** Nb_4_AlC_3_ MAX, **c)** ML Nb_2_CT*_x_* MXene, **d)** ML Nb_4_C_3_T*_x_* MXene, **e)** SL Nb_2_CT*_x_* MXene, and **f)** SL Nb_4_C_3_T*_x_* MXene.


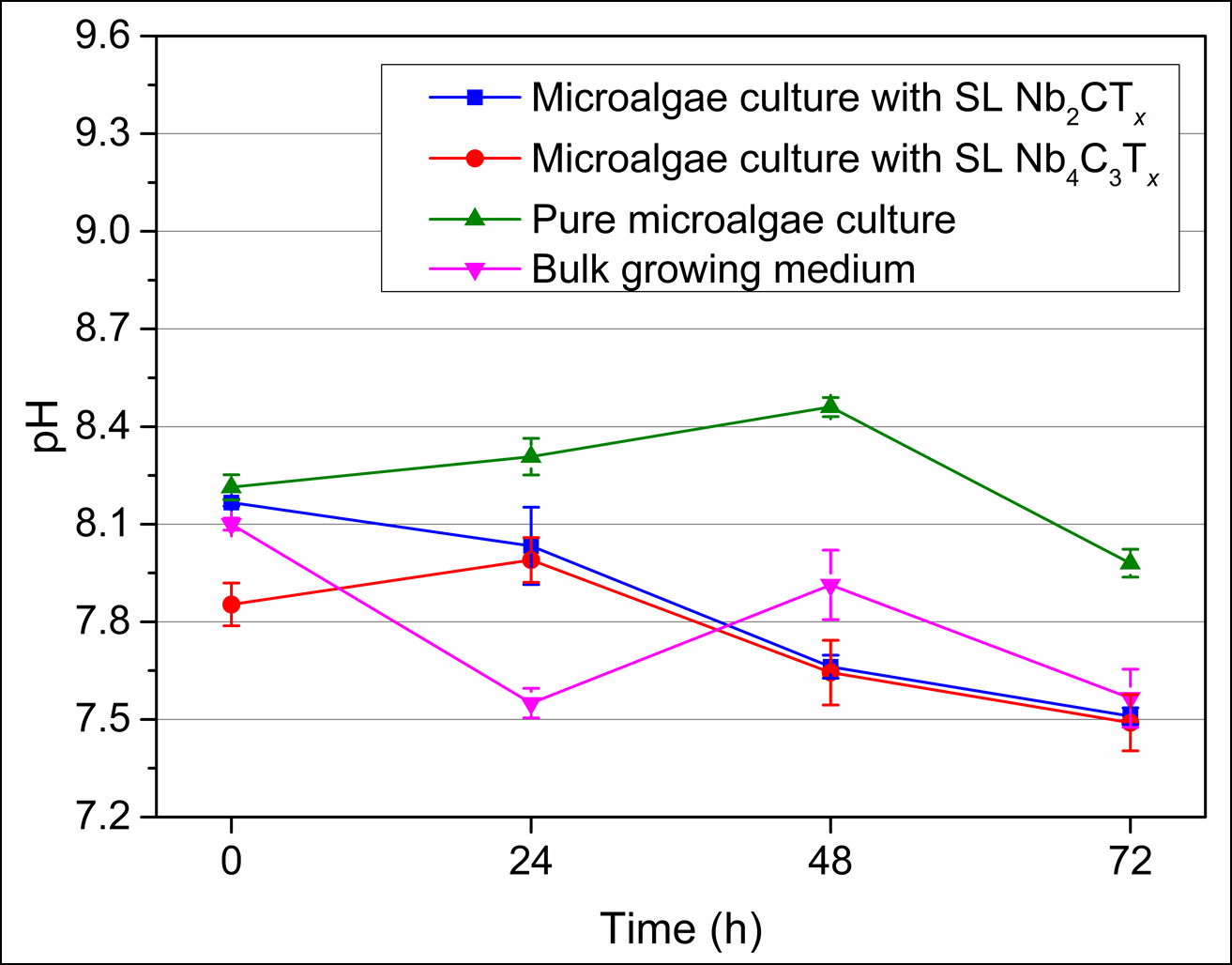


**Figure S5.** pH changes over time measured for microalgae cultures incubated in the presence of SL Nb_2_CT*_x_* and Nb_4_C_3_T*_x_*, as well for pure microalgae culture and bulk growing medium.


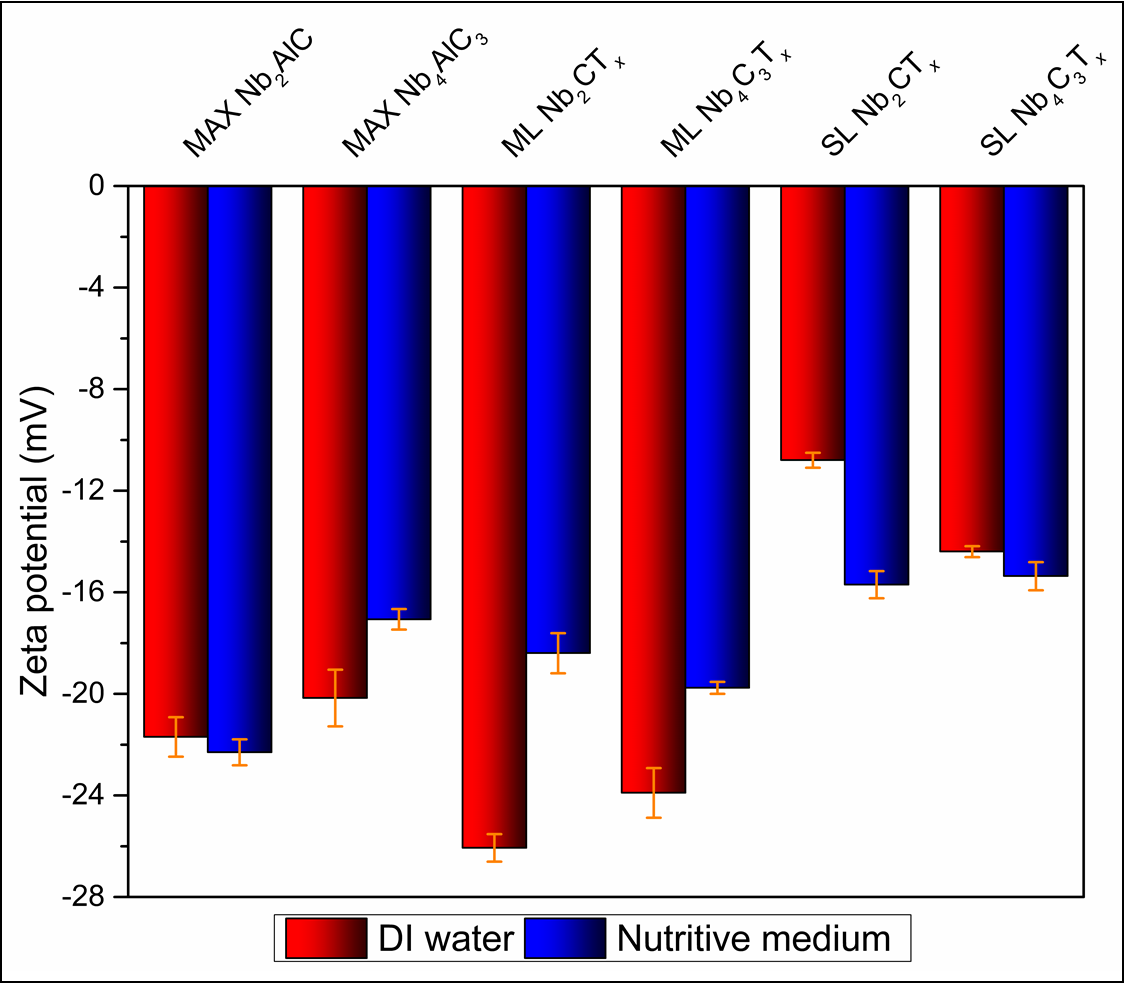


**Figure S6.** Dispersion stability of Nb-MAX phases and Nb-MXenes in DI-water as well as nutritive medium for microalgae cultivation.


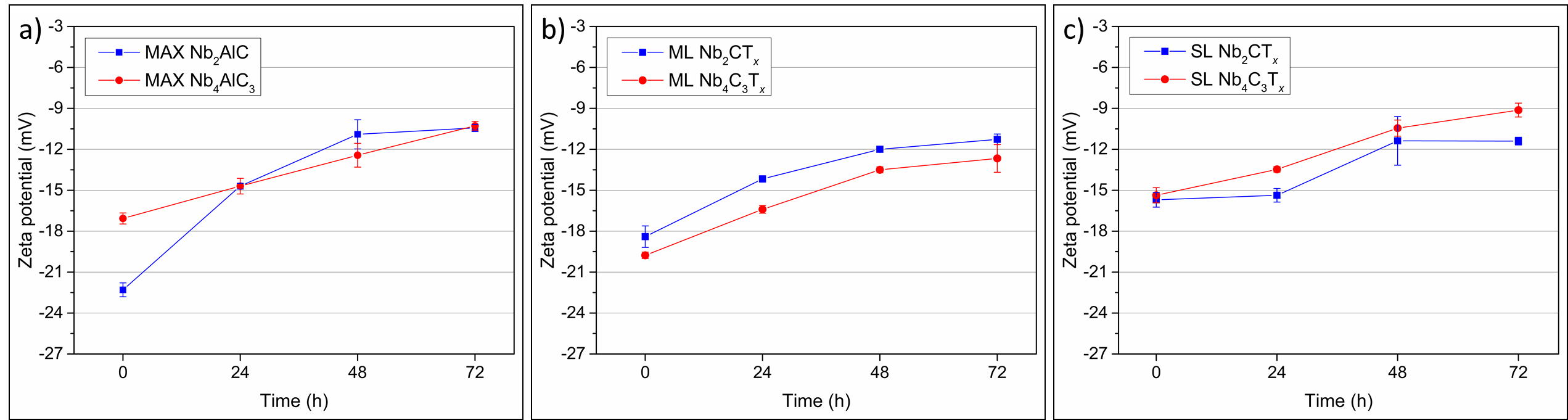


**Figure S7.** Dispersion stability of **a)** Nb-MAX phases, **b)** ML Nb-MXenes, and **c)** SL Nb-MXenes in nutritive medium for microalgae cultivation over time.


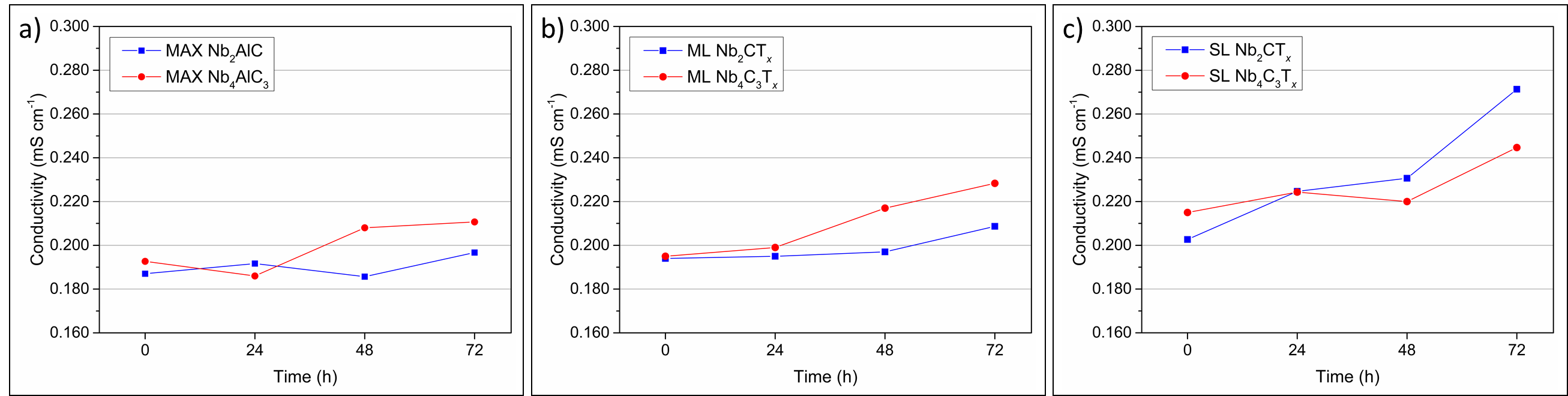


**Figure S8.** Conductivity of **a)** Nb-MAX phases, **b)** ML Nb-MXenes, and **c)** SL Nb-MXenes in nutritive medium for microalgae cultivation measured over time.


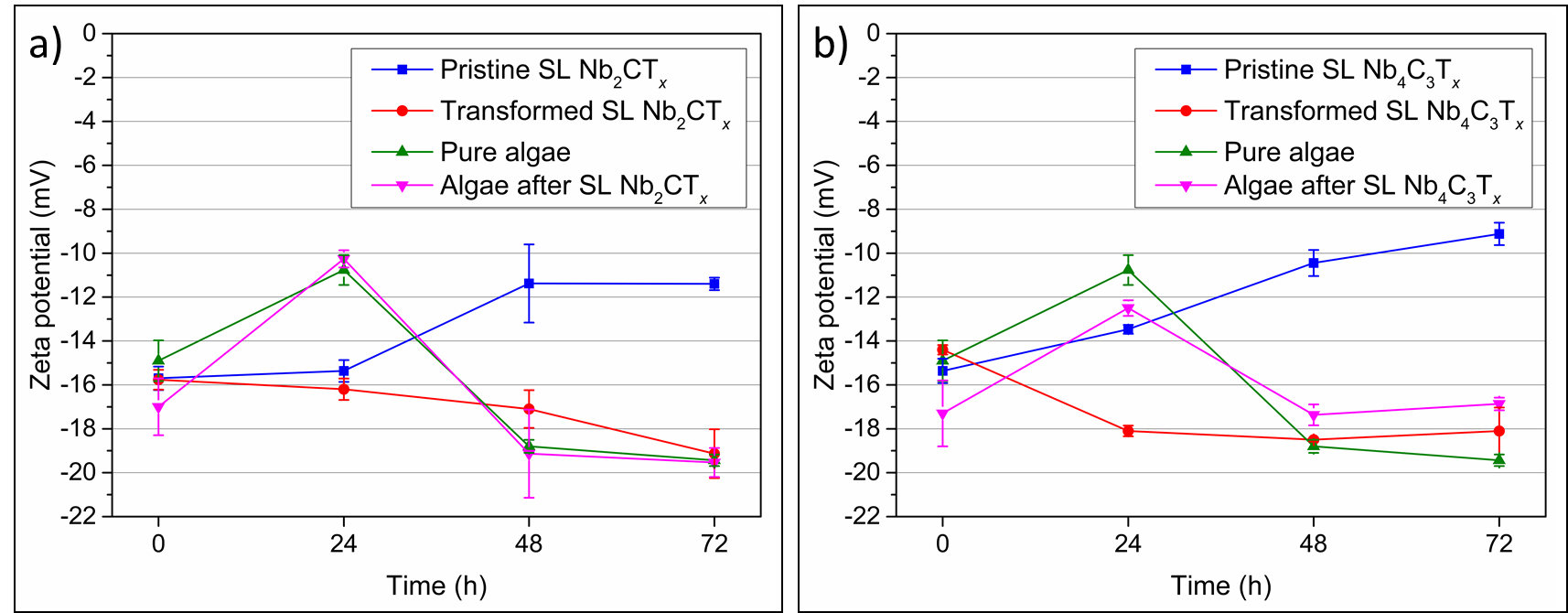


**Figure S9.** Analysis of dispersion stability of SL **a)** Nb_2_CT*_x_* and **b)** Nb_4_C_3_T*_x_* MXenes after interacting with microalgae in nutritive medium and cultivated over time. For this purpose, reference microalgae samples were analyzed as well as the pristine SL Nb_2_CT*_x_* and SL Nb_4_C_3_T*_x_* MXenes, SL Nb_2_CT*_x_* and SL Nb_4_C_3_T*_x_* MXenes transformed by microalgae and microalgae after treatment with SL Nb_2_CT*_x_* and SL Nb_4_C_3_T*_x_* MXenes.


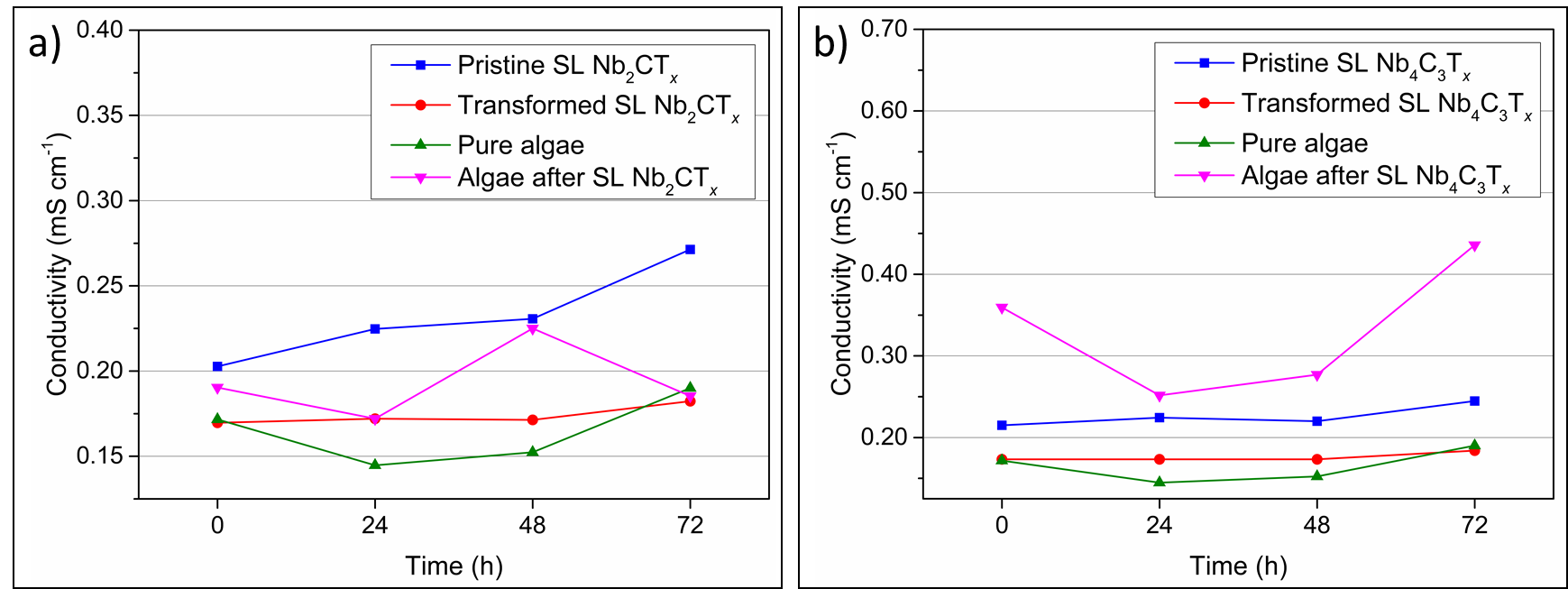


**Figure S10.** Analysis of conductivity of SL **a)** Nb_2_CT*_x_* and **b)** Nb_4_C_3_T*_x_* MXenes after interacting with microalgae in nutritive medium and cultivated over time. For this purpose, reference microalgae samples were analyzed as well as the pristine SL Nb_2_CT*_x_* and SL Nb_4_C_3_T*_x_* MXenes, SL Nb_2_CT*_x_* and SL Nb_4_C_3_T*_x_* MXenes transformed by microalgae and microalgae after treatment with SL Nb_2_CT*_x_* and SL Nb_4_C_3_T*_x_* MXenes.


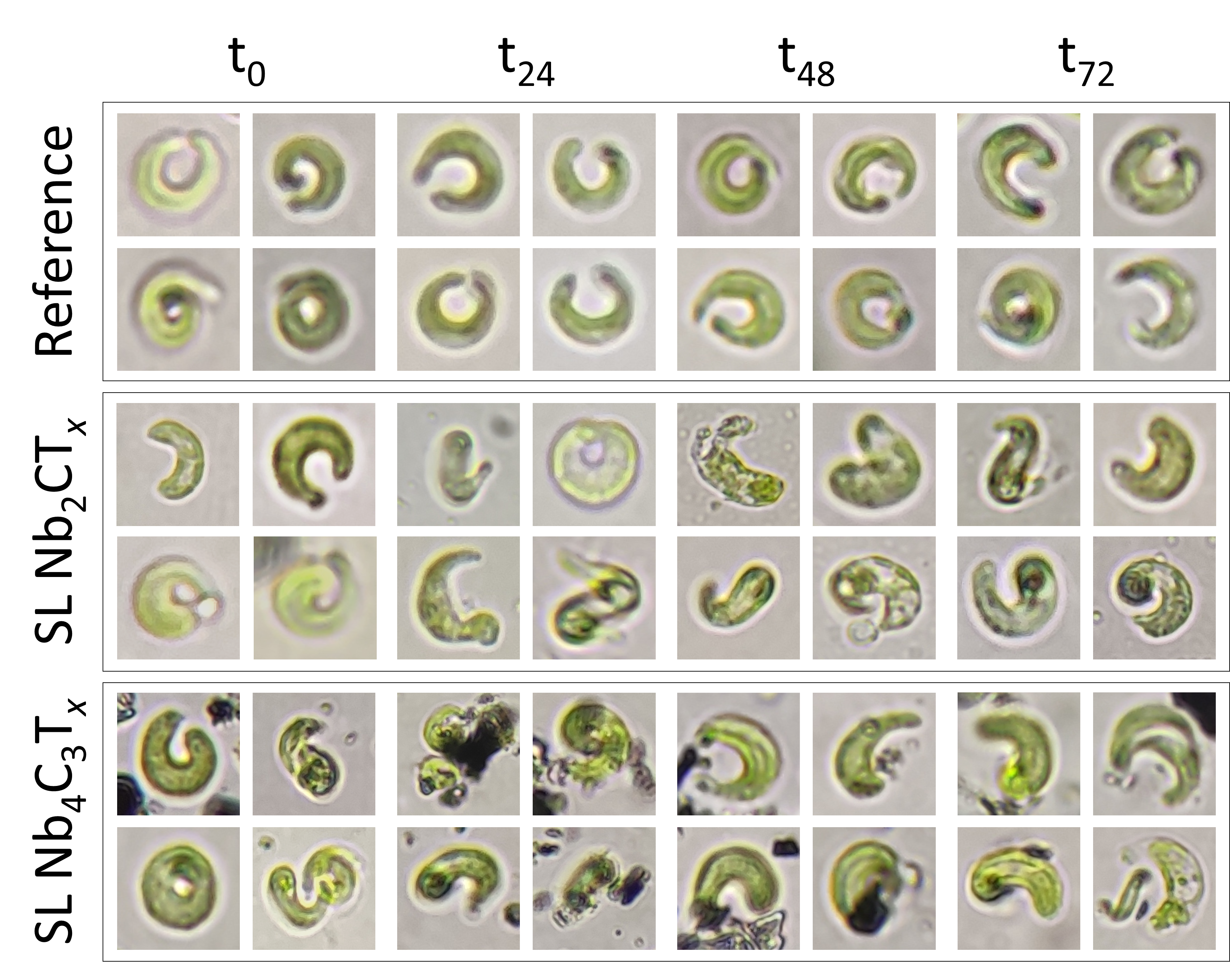


**Figure S11.** Set of microscopic images revealing microalgae morphological changes after interaction with SL Nb_2_CT*_x_* and Nb_4_C_3_T*_x_* MXenes in comparison with untreated green microalgae.


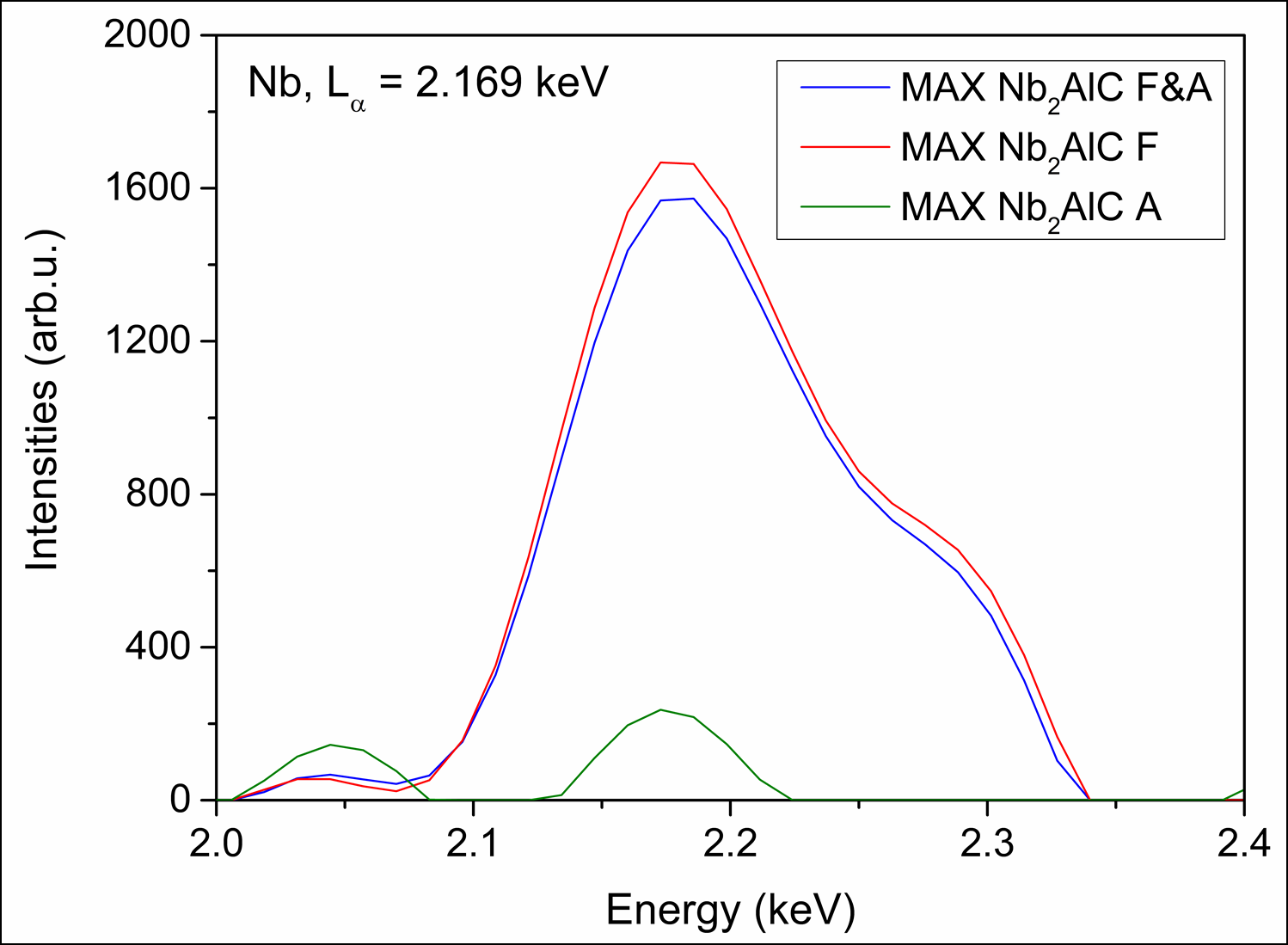


**Figure S12.** XRF spectra of niobium peak area for samples interacting with Nb_2_AlC MAX phase. Analysis compares complete microalgae+MAX mixture **(F&A**, marked with blue**)**, separated MAX **(F**, marked with red**)**, and microalgae separated from interacting MAX phase (**A**, marked with green).


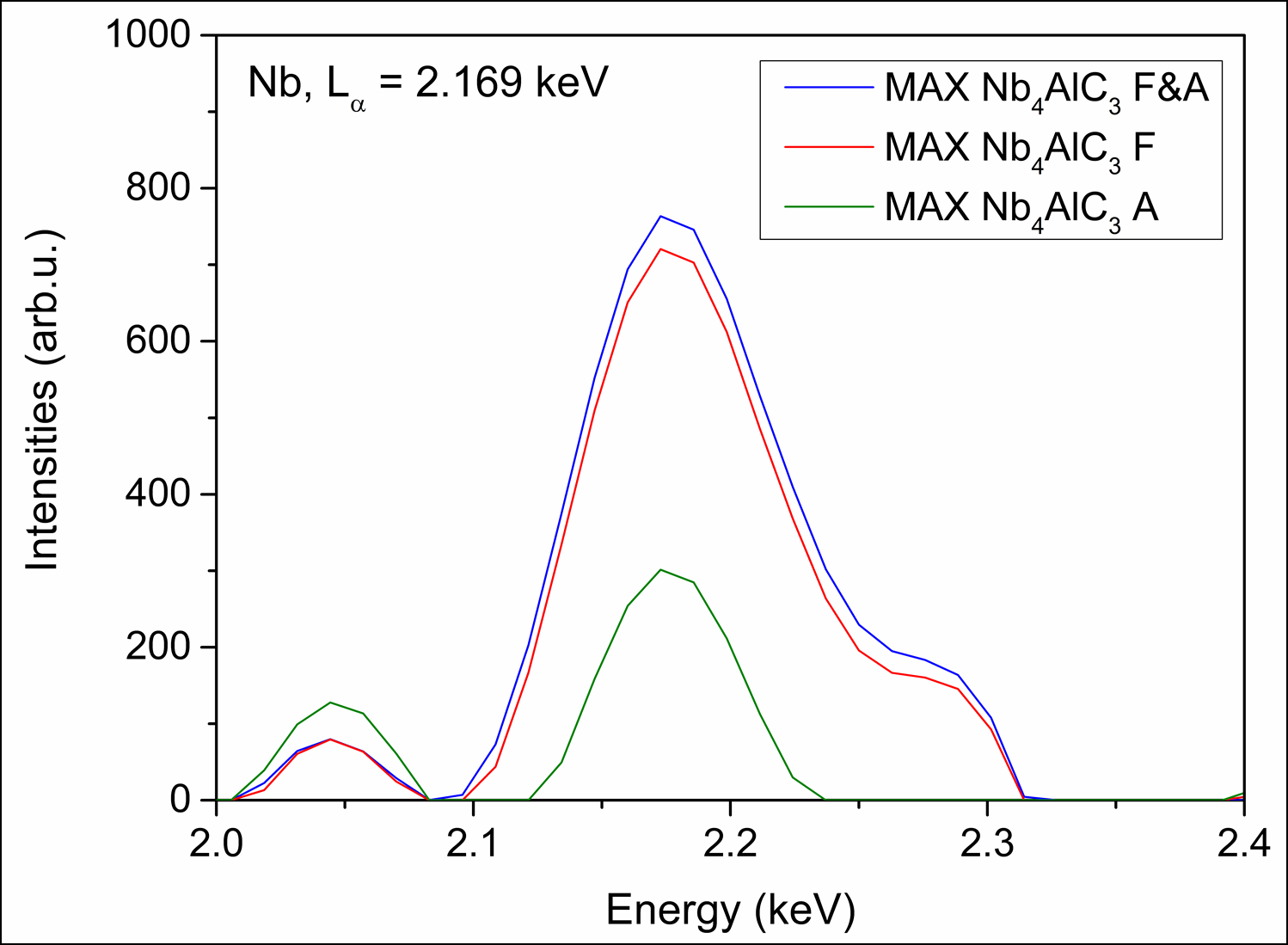


**Figure S13.** XRF spectra of niobium peak area for samples interacting with Nb_4_AlC_3_ MAX phase. Analysis compares complete microalgae+MAX mixture **(F&A**, marked with blue**)**, separated MAX **(F**, marked with red**)**, and microalgae separated from interacting MAX phase (**A**, marked with green).


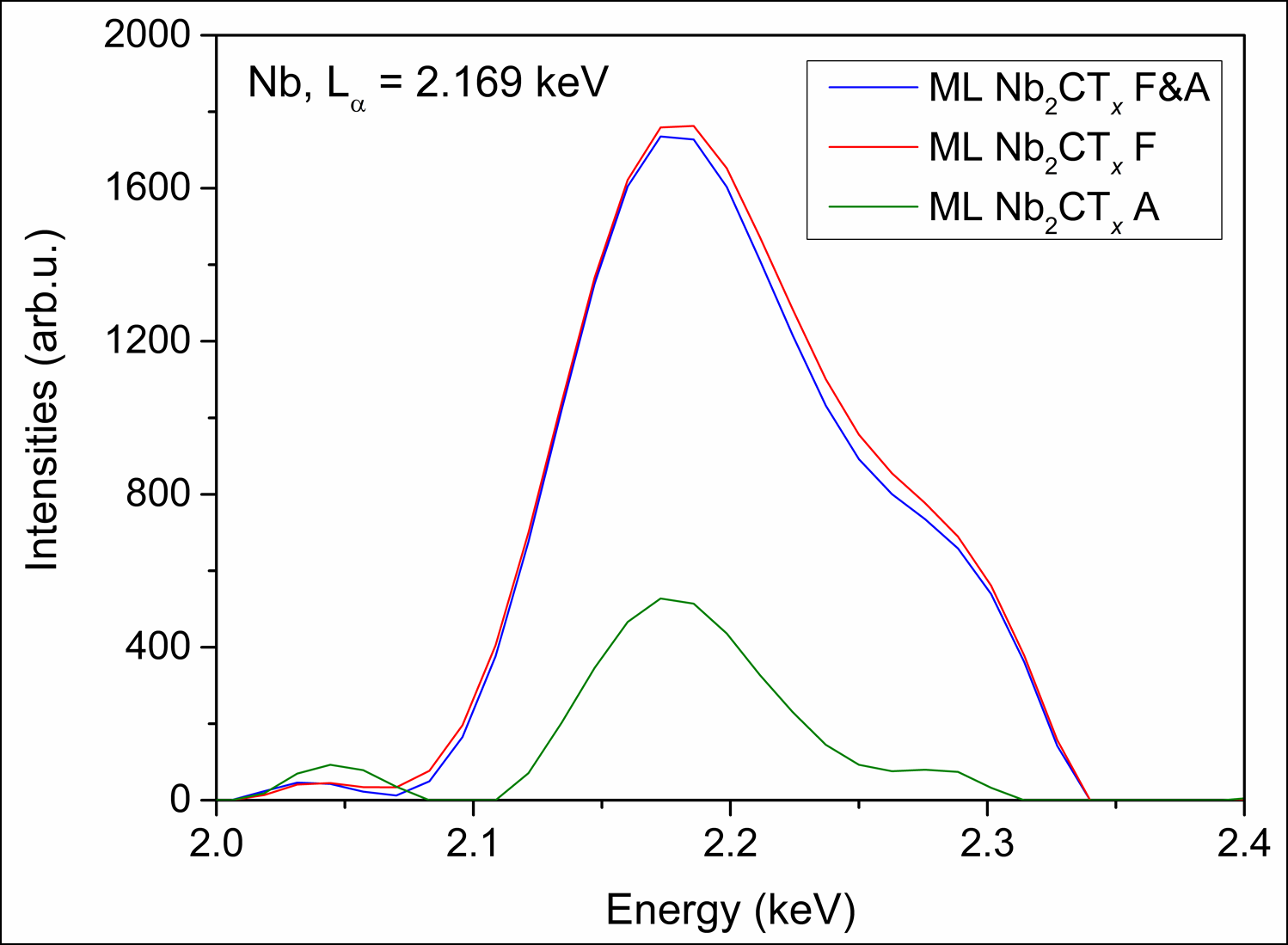


**Figure S14.** XRF spectra of niobium peak area for samples interacting with ML Nb_2_CT*_x_* MXene. Analysis compares complete microalgae+MXene mixture **(F&A**, marked with blue**)**, separated MXene **(F**, marked with red**)**, and microalgae separated from interacting MXene (**A**, marked with green).


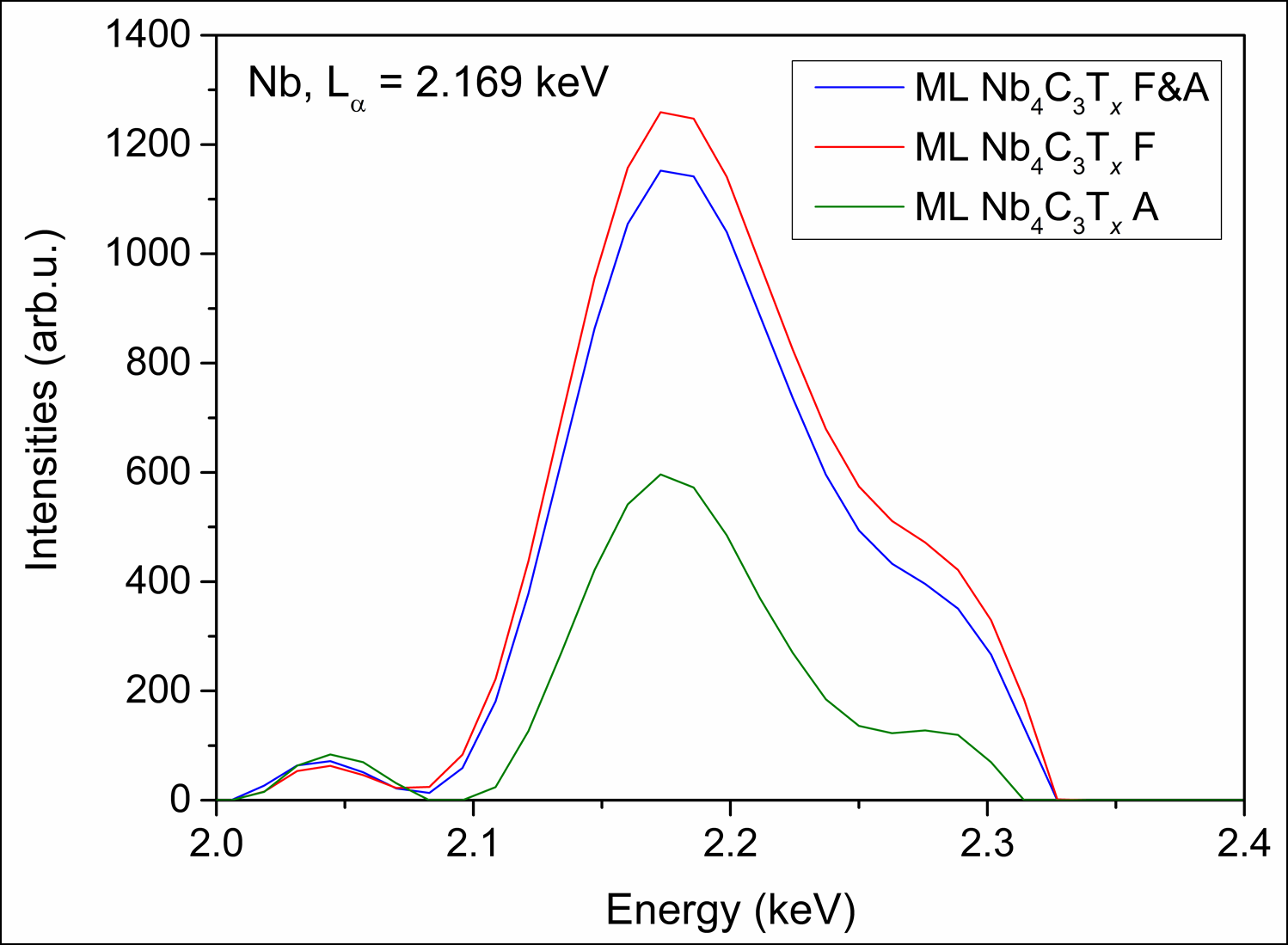


**Figure S15.** XRF spectra of niobium peak area for samples interacting with ML Nb_4_C_3_T_x_ MXene. Analysis compares complete microalgae+MXene mixture **(F&A**, marked with blue**)**, separated MXene **(F**, marked with red**)**, and microalgae separated from interacting MXene (**A**, marked with green).


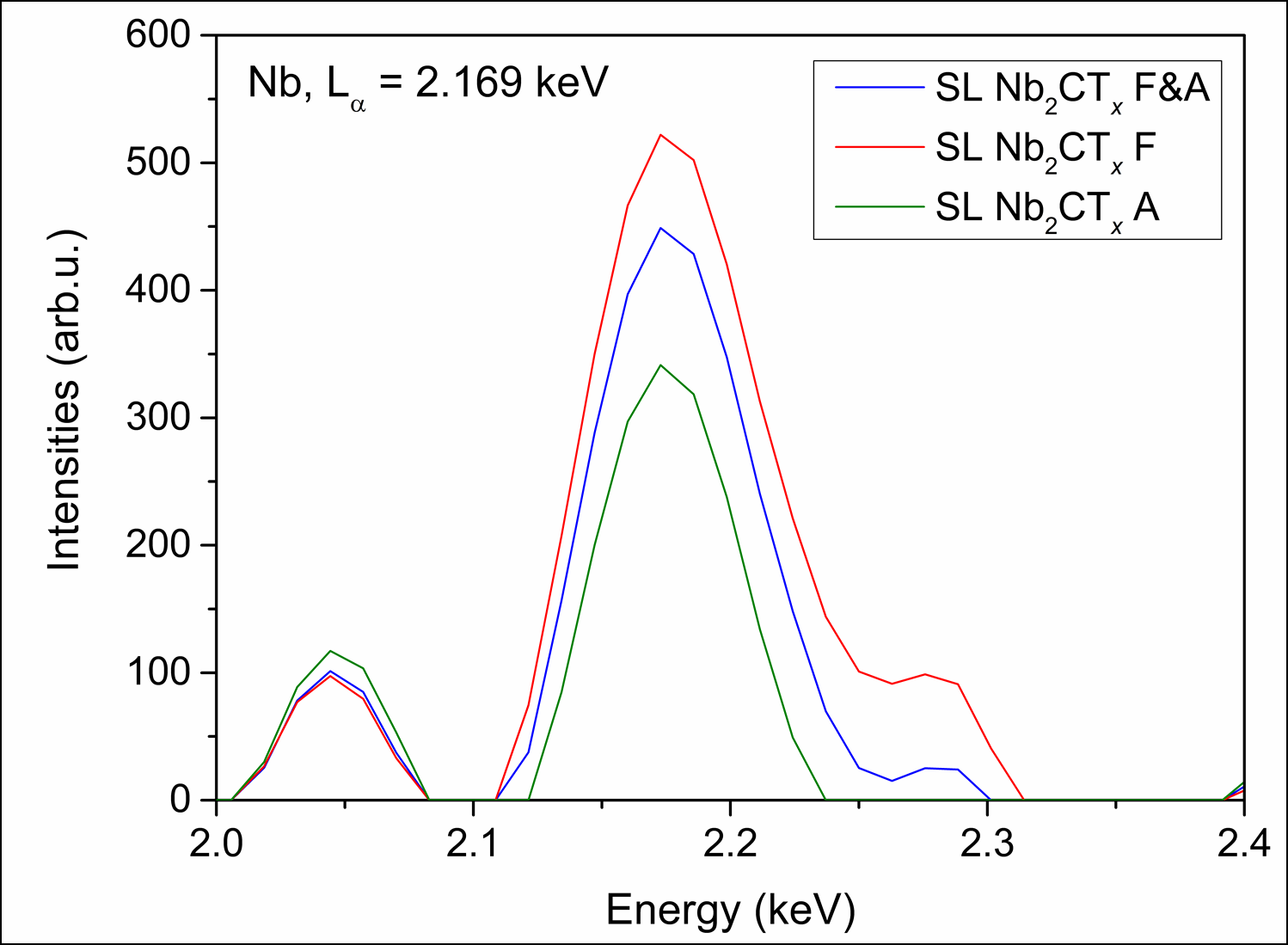


**Figure S16.** XRF spectra of niobium peak area for samples interacting with SL Nb_2_CT*_x_* MXene. Analysis compares complete microalgae+MXene mixture **(F&A**, marked with blue**)**, separated MXene **(F**, marked with red**)**, and microalgae separated from interacting MXene (**A**, marked with green).


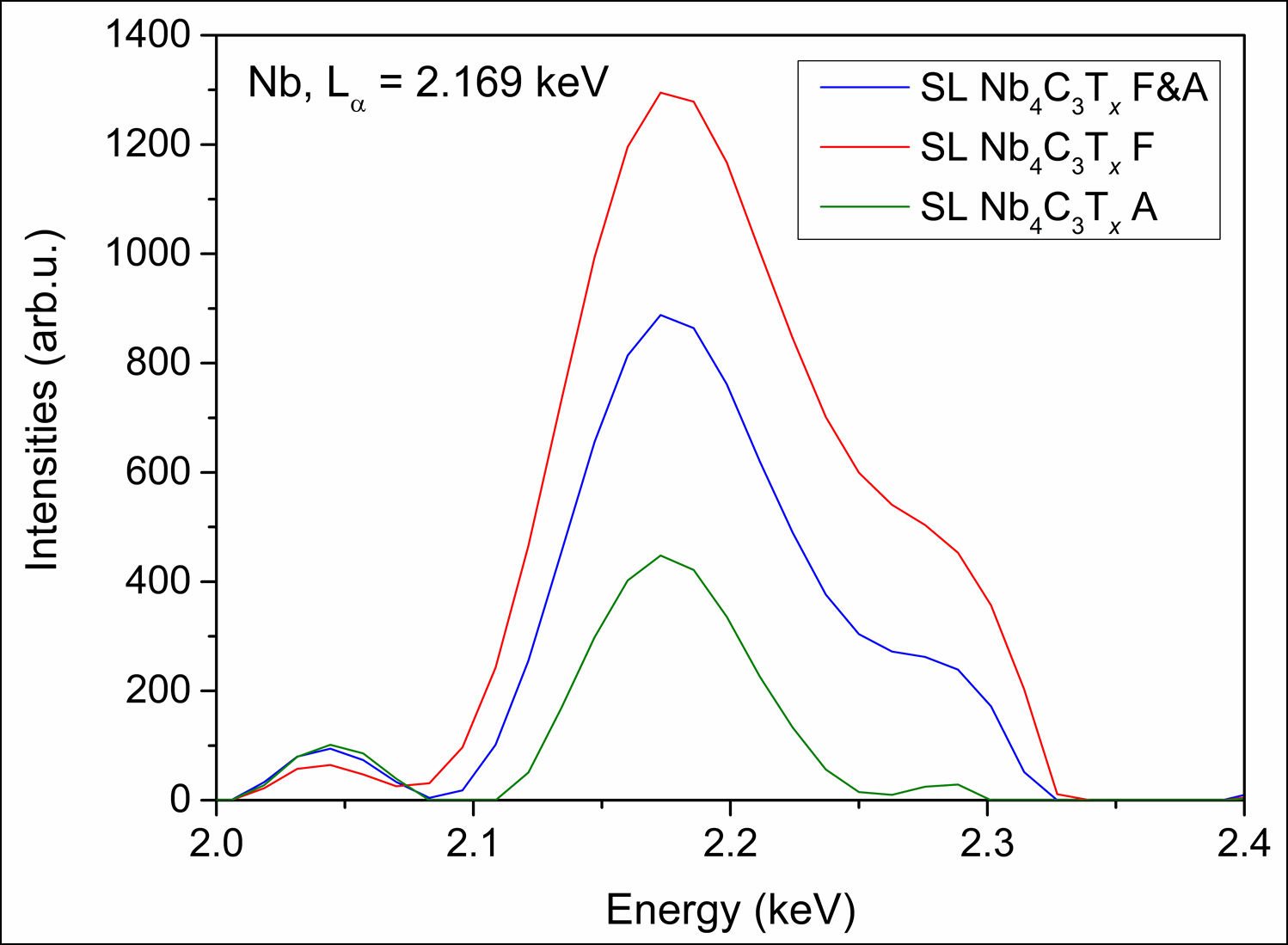


**Figure S17.** XRF spectra of niobium peak area for samples interacting with SL Nb_4_C_3_T*_x_* MXene. Analysis compares complete microalgae+MXene mixture **(F&A**, marked with blue**)**, separated MXene **(F**, marked with red**)**, and microalgae separated from interacting MXene (**A**, marked with green).


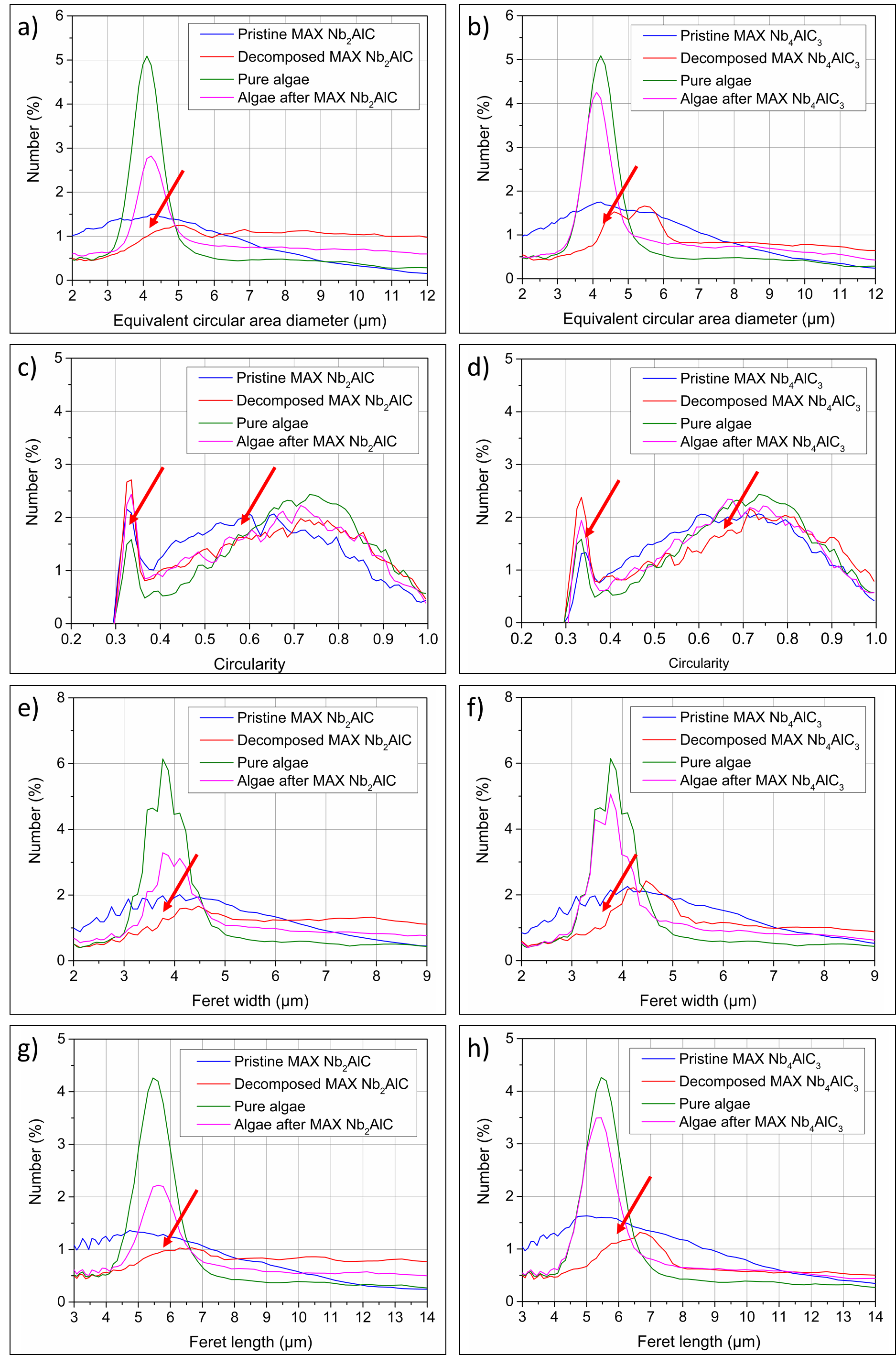


**Figure S18.** MAX transformation analysis after interacting with microalgae. The dynamic particle shape analysis considered parameters such as **a-b)** equivalent circular area diameter, **c-d)** circularity, **e-f)** Feret width and **g-h)** Feret length. For this purpose, two reference microalgae samples were analyzed as well as the pristine Nb_2_AlC and Nb_4_AlC_3_ MAX phases, Nb_2_AlC and Nb_4_AlC_3_ MAX phases decomposed by microalgae and microalgae after treatment with Nb_2_AlC and Nb_4_AlC_3_ MAX phases. The red arrows indicate transformation in investigated shape parameters of 2D nanoflakes.


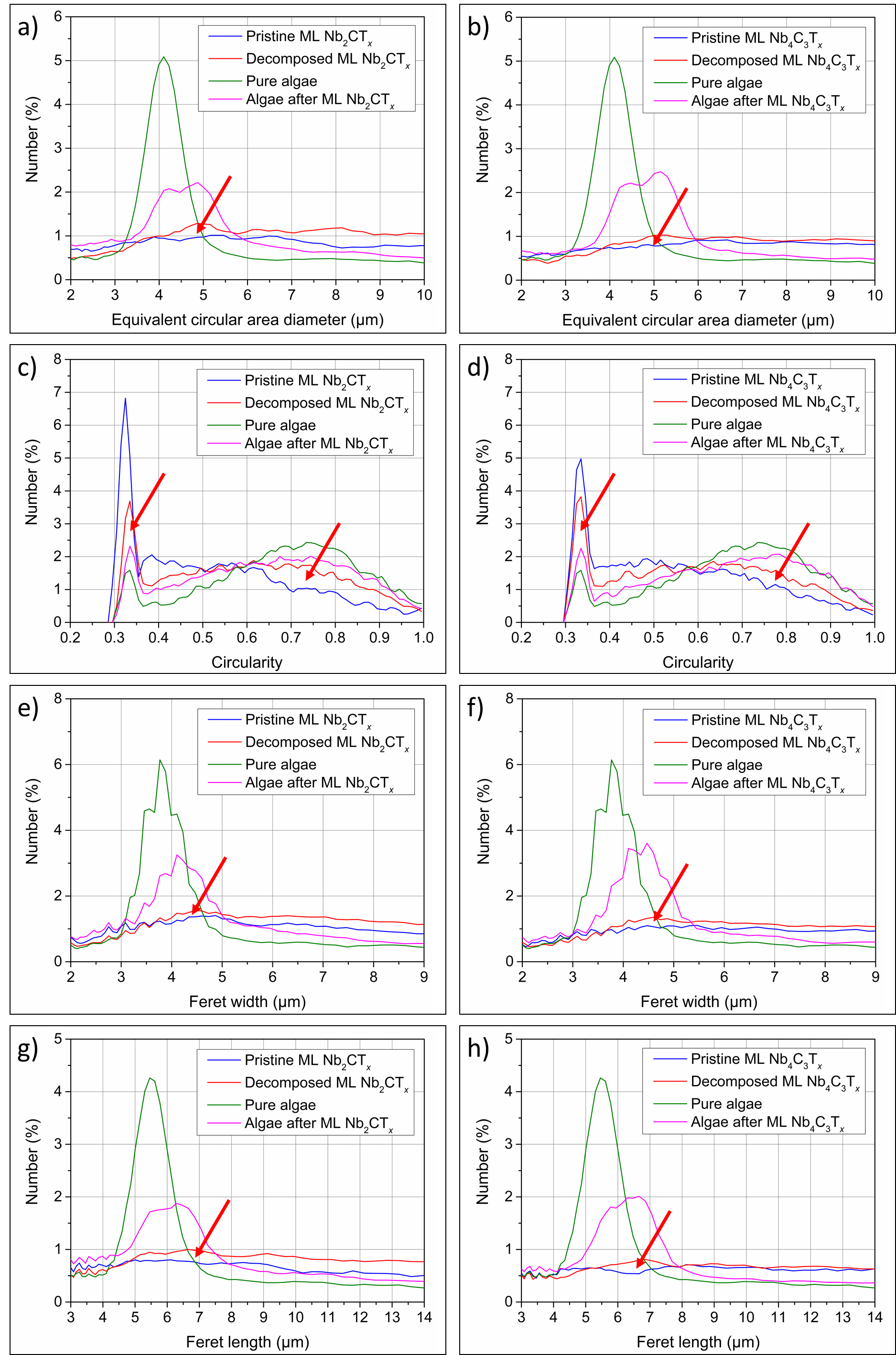


**Figure S19.** ML transformation analysis after interacting with microalgae. The dynamic particle shape analysis considered parameters such as **a-b)** equivalent circular area diameter, **c-d)** circularity, **e-f)** Feret width and **g-h)** Feret length. For this purpose, two reference microalgae samples were analyzed as well as the pristine ML Nb_2_CT*_x_* and ML Nb_4_C_3_T*_x_* MXenes, ML Nb_2_CT*_x_* and ML Nb_4_C_3_T*_x_* MXenes decomposed by microalgae and microalgae after treatment with ML Nb_2_CT*_x_* and ML Nb_4_C_3_T*_x_* MXenes. The red arrows indicate transformation in investigated shape parameters of 2D nanoflakes.


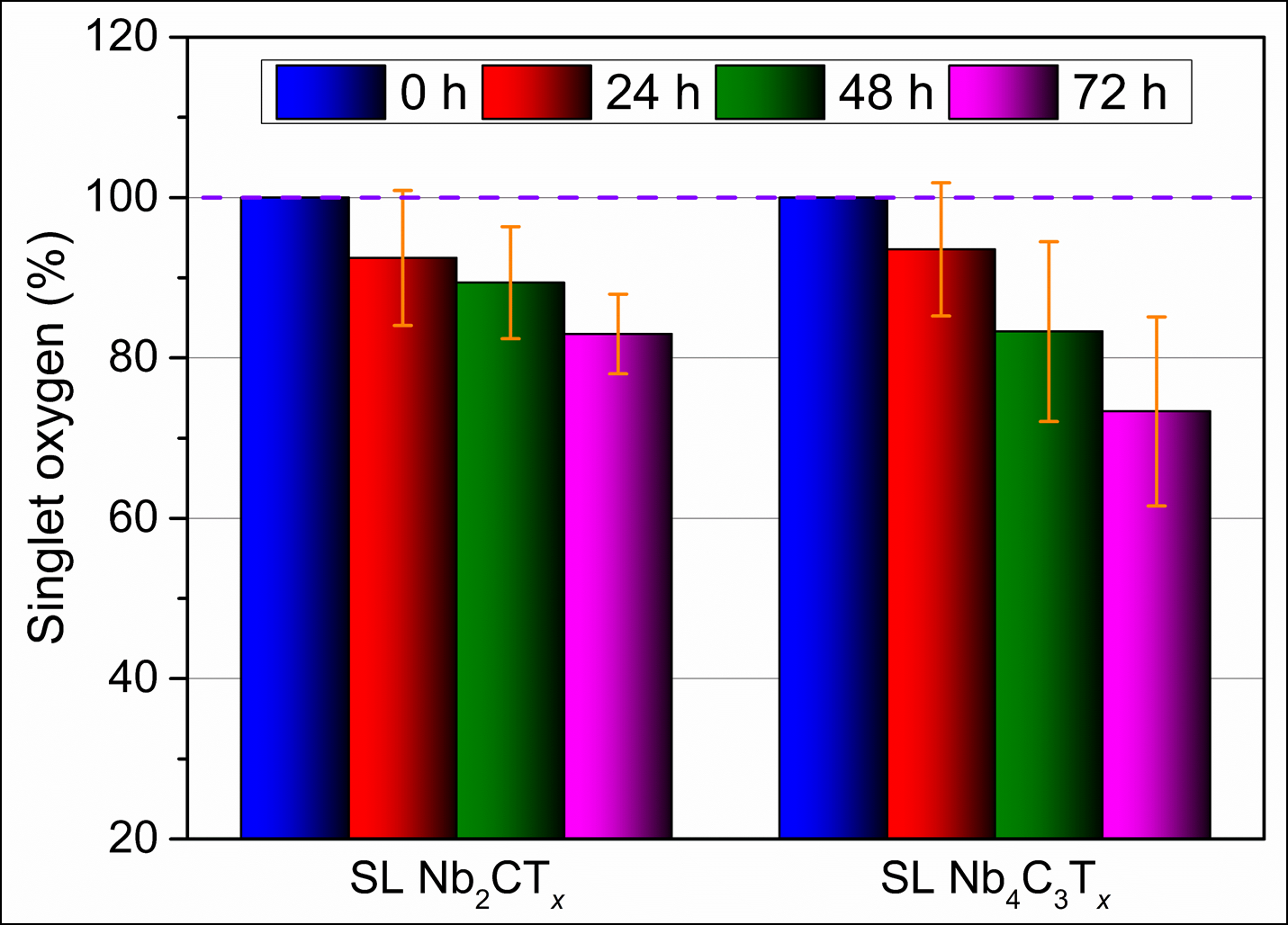


**Figure S20.** Level of singlet oxygen detected for the microalgae cultures incubated in the presence of SL Nb_2_CT*_x_* and Nb_4_C_3_T*_x_* at the following time points. We marked the reference ROS level with a purple line.


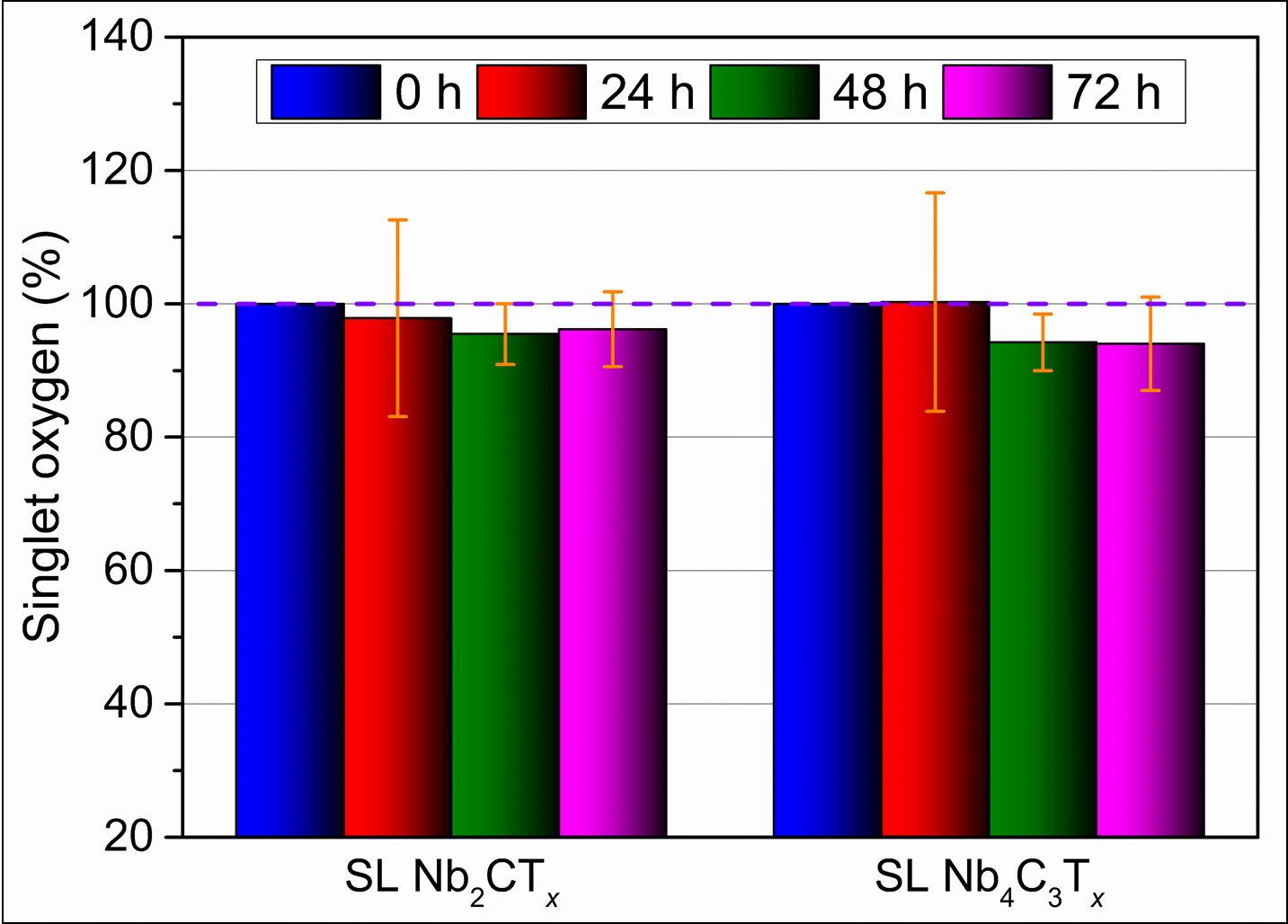


**Figure S21.** Level of singlet oxygen detected in control assay of SL Nb_2_CT*_x_* and Nb_4_C_3_T*_x_* MXenes incubated without the presence of green microalgae.


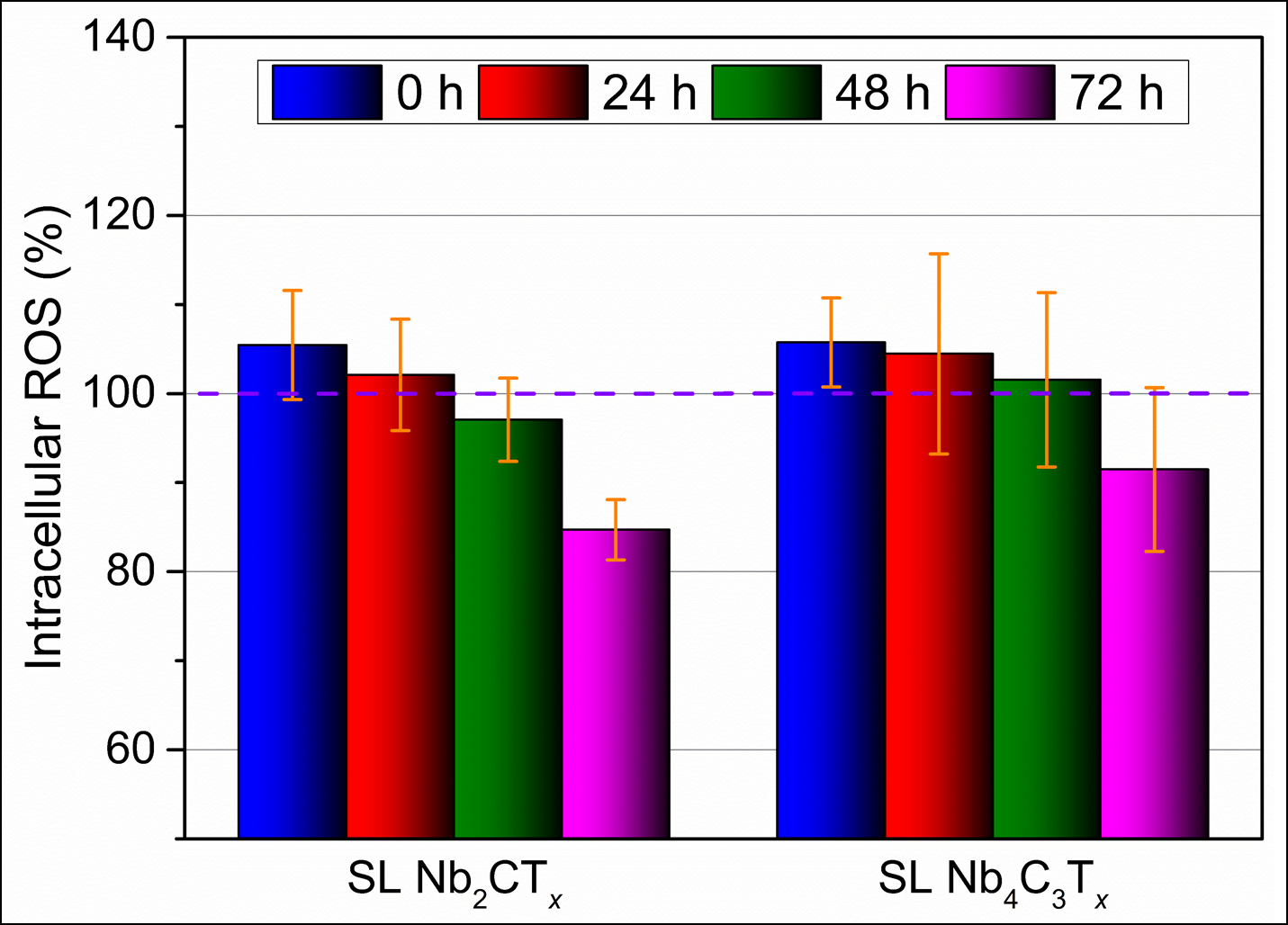


**Figure S22.** Level of intercellular reactive oxygen species detected for the microalgae cultures incubated in the presence of SL Nb_2_CT*_x_* and Nb_4_C_3_T*_x_* at the following time points. We marked the reference ROS level with a purple line.


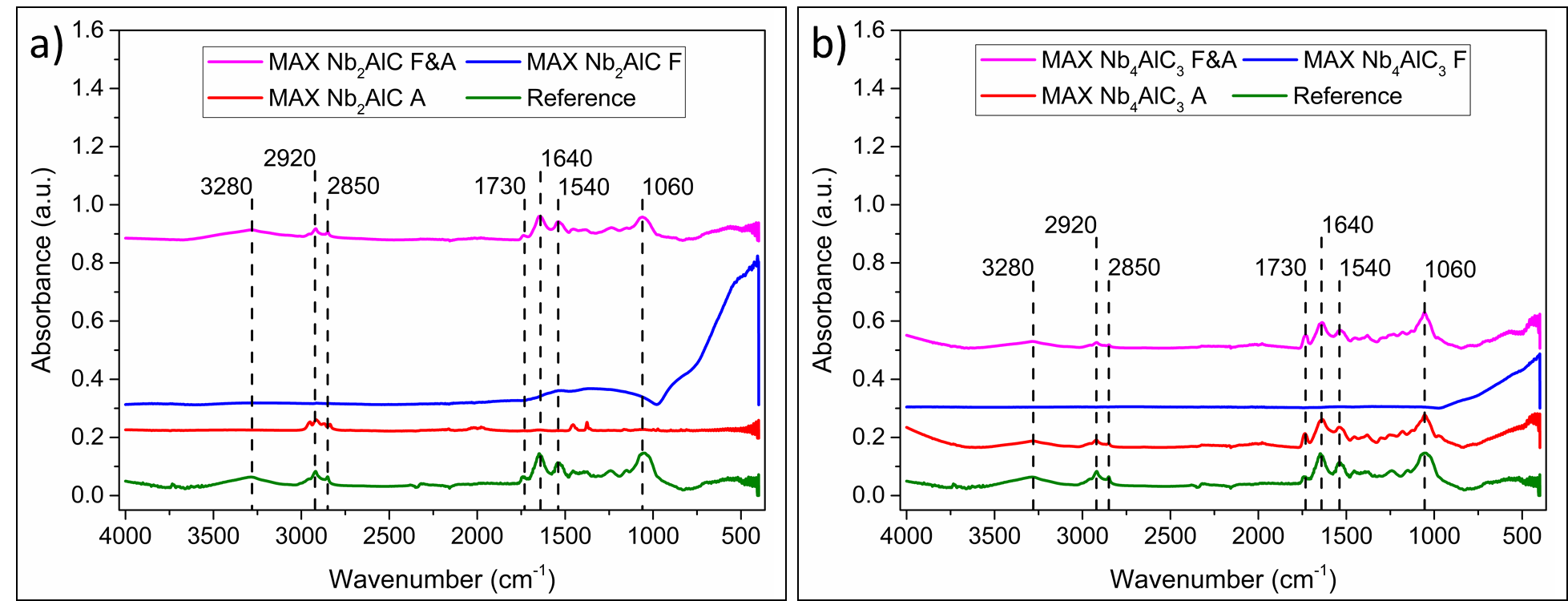


**Figure S23.** FT-IR analysis for microalgae incubated with MAX phases (F&A), separate nanoflakes (F), and microalgae separated from incubations with MAX phases (A) for: **a)** MAX Nb_2_AlC and **b)** MAX Nb_4_AlC_3_.


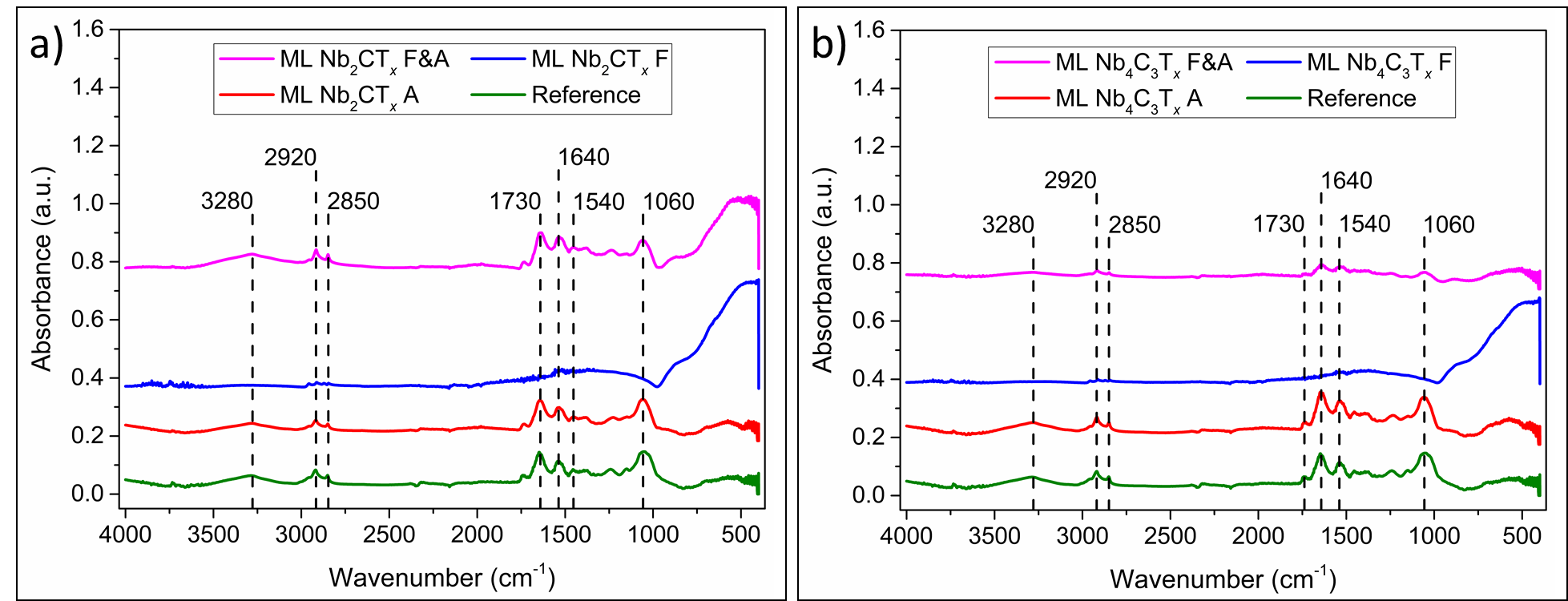


**Figure S24.** FT-IR analysis for microalgae incubated with single-layer MXene nanoflakes (F&A), separate nanoflakes (F), and microalgae separated from incubations with nanoflakes (A) for: **a)** ML Nb_2_CT*_x_* and **b)** ML Nb_4_C_3_T*_x_*. For simplicity, the T*_x_* annotation was omitted in the image.


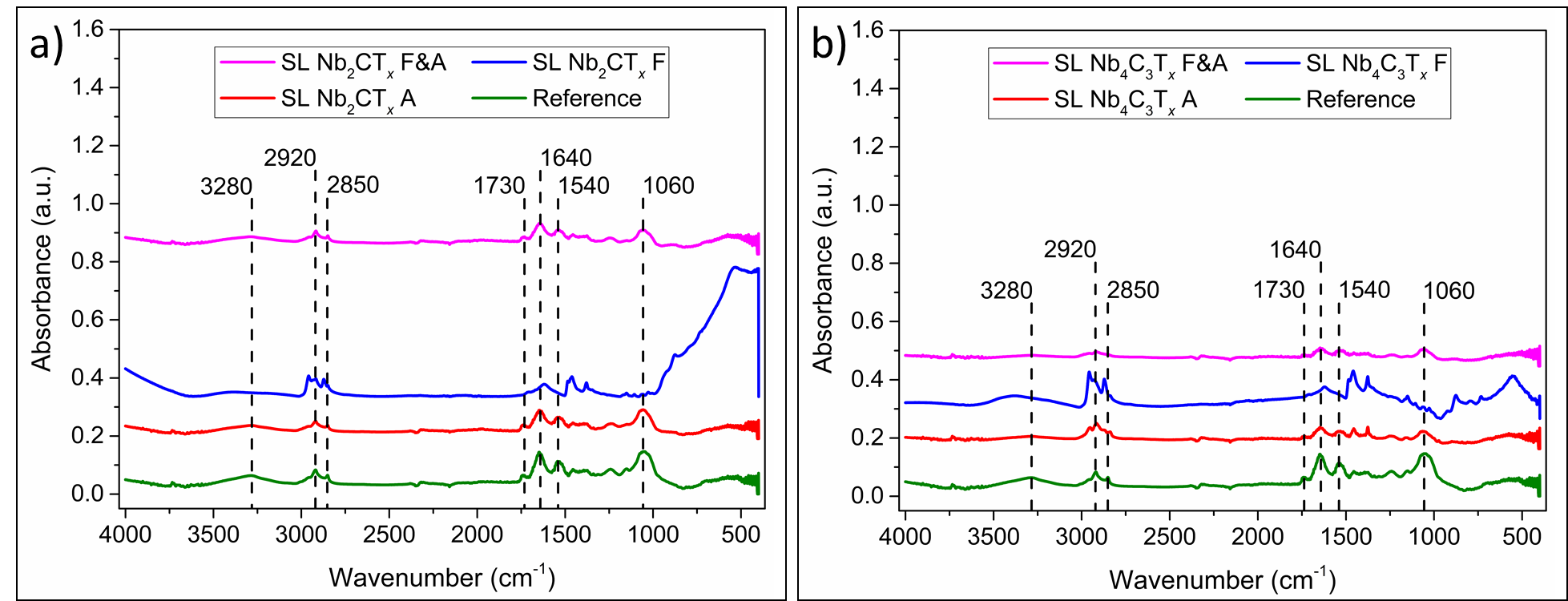


**Figure S25.** FT-IR analysis for microalgae incubated with single-layer MXene nanoflakes (F&A), separate nanoflakes (F), and microalgae separated from incubations with nanoflakes (A) for: **a)** SL Nb_2_CT*_x_* and **b)** SL Nb_4_C_3_T*_x_*. For simplicity, the T*_x_* annotation was omitted in the image.

**Table S1**. Individual signals obtained from XPS analysis, comprising the chemical state of elements for microalgae growing in the presence of single-layer Nb_2_CT*_x_*, and after MXenes separation.

| **Region** | **Binding energy (eV)** | **FWHM (eV)** | **Fraction** | **Assigned to** | **Ref.** |
| --- | --- | --- | --- | --- | --- |
| **Nb 3d_5/2_ (3d_3/2_)** | 207.2 (209.9) | 1.5 (1.5) | 1 | Nb_2_O_5_ | 1, 2 |
| **C 1s** | 284.8 | 1.8 | 0.76 | C-C | 1 |
|  | 286.3 | 1.8 | 0.15 | CH_x_/CO | 1, 2, 3, 4 |
|  | 287.9 | 1.8 | 0.07 | C=O | 3, 4 |
|  | 289.4 | 1.8 | 0.02 | -COOH | 3, 4 |
| **O 1s** | 530.5 | 1.7 | 0.08 | Nb_2_O_5_ | 1, 2 |
|  | 531.7 | 1.6 | 0.21 | C=O | 3, 5 |
|  | 532.7 | 1.6 | 0.40 | C-O | 3, 5 |
|  | 533.3 | 1.9 | 0.31 | H_2_O_ads_ | 3, 5 |

**Table S2**. Individual signals obtained from XPS analysis, comprising the chemical state of elements for microalgae growing in the presence of single-layer Nb_4_C_3_T*_x_*, and after MXenes separation.

| **Region** | **Binding energy (eV)** | **FWHM (eV)** | **Fraction** | **Assigned to** | **Ref.** |
| --- | --- | --- | --- | --- | --- |
| **Nb 3d_5/2_ (3d_3/2_)** | 207.1 (209.8) | 1.6 (1.6) | 1 | Nb_2_O_5_ | 1, 2 |
| **C 1s** | 284.8 | 1.4 | 0.68 | C-C | 1 |
|  | 286.2 | 1.4 | 0.21 | CH_x_/CO | 1, 2, 3, 4 |
|  | 287.6 | 1.4 | 0.07 | C=O | 3, 4 |
|  | 289.0 | 1.4 | 0.04 | -COOH | 3, 4 |
| **O 1s** | 530.6 | 1.6 | 0.05 | Nb_2_O_5_ | 1, 2 |
|  | 531.6 | 1.6 | 0.30 | C=O | 3, 5 |
|  | 532.6 | 1.6 | 0.45 | C-O | 3, 5 |
|  | 533.6 | 1.9 | 0.20 | H_2_O_ads_ | 3, 5 |

**Table S3.** Summary of elemental global at.%, including non MXene entities, for microalgae growing in the presence of single-layer Nb-MXenes, and after separation of nanoflakes.

|  | **Nb** | **C** | **O** | **N** | **Ca** | **S** | **Si** |
| --- | --- | --- | --- | --- | --- | --- | --- |
| **Nb_2_CT*_x_*** | 3.91 | 59.23 | 28.94 | 2.14 | 0.83 | 1.37 | 3.59 |
| **Nb_4_C_3_T*_x_*** | 5.45 | 52.56 | 34.32 | 3.14 | 0.64 | 0.85 | 3.04 |

**Table S4**. Individual signals obtained from XPS analysis, comprising the chemical state of elements for microalgae growing in the presence of single-layer Nb_2_CT*_x_* MXene.

| **Region** | **Binding energy (eV)** | **FWHM (eV)** | **Fraction** | **Assigned to** | **Ref.** |
| --- | --- | --- | --- | --- | --- |
| **Nb 3d_5/2_ (3d_3/2_)** | 205.7 (208.4) | 0.7 (0.8) | 0.15 | Nb^3+^-O | 1, 2 |
|  | 207.5 (210.2) | 1.5 (1.5) | 0.85 | Nb_2_O_5_ | 1, 6 |
| **C 1s** | 284.8 | 1.5 | 0.58 | C-C | 1, 2, 3, 4 |
|  | 286.1 | 1.5 | 0.25 | CH_x_/C-O | 3, 4 |
|  | 287.4 | 1.5 | 0.11 | C=O | 3, 4 |
|  | 288.8 | 1.5 | 0.06 | -COOH | 3, 4 |
| **O 1s** | 530.6 | 1.5 | 0.24 | Nb_2_O_5_ | 1, 2 |
|  | 531.5 | 1.5 | 0.17 | C=O | 3, 5 |
|  | 532.4 | 1.5 | 0.26 | C-O | 3, 5 |
|  | 533.3 | 1.7 | 0.32 | H_2_O_ads_ | 3, 5 |

**Table S5**. Individual signals obtained from XPS analysis, comprising the chemical state of elements for microalgae growing in the presence of single-layer Nb_4_C_3_T*_x_* MXene.

| **Region** | **Binding energy (eV)** | **FWHM (eV)** | **Fraction** | **Assigned to** | **Ref.** |
| --- | --- | --- | --- | --- | --- |
| **Nb 3d_5/2_ (3d_3/2_)** | 203.0 (205.7) | 1.2 (0.8) | 0.09 | Nb-C | 1, 2 |
|  | 207.4 (210.1) | 1.5 (1.5) | 0.91 | Nb_2_O_5_ | 1, 6 |
| **C 1s** | 282.3 | 0.9 | 0.01 | Nb-C | 1 |
|  | 284.8 | 1.6 | 0.52 | C-C | 1, 2, 3, 4 |
|  | 286.4 | 1.6 | 0.29 | CH_x_/C-O | 3, 4 |
|  | 287.7 | 1.6 | 0.12 | C=O | 3, 4 |
|  | 288.9 | 1.6 | 0.06 | -COOH | 3, 4 |
| **O 1s** | 530.6 | 1.5 | 0.31 | Nb_2_O_5_ | 1, 2 |
|  | 531.5 | 1.4 | 0.18 | C=O | 3, 5 |
|  | 532.4 | 1.6 | 0.23 | C-O | 3, 5 |
|  | 533.3 | 1.9 | 0.28 | H_2_O_ads_ | 3, 5 |

**Table S6.** Mass concentrations of nutrients in the microalgal culture medium.

| **Stock solution** | **Nutrient** | **Mass concentration in stock solution** | **Final mass concentration in test solution** |
| --- | --- | --- | --- |
| **1: Macronutrients** | NH_4_Cl | 1.5 g L^-1^ | 15 mg L^-1^ (N: 3.9 mg L^-1^) |
|  | MgCl_2_⋅6H_2_O | 1.2 g L^-1^ | 12 mg L^-1^ (Mg: 2.9 mg L^-1^) |
|  | CaCl_2_⋅2H_2_O | 1.8 g L^-1^ | 18 mg L^-1^ (Ca: 4.9 mg L^-1^) |
|  | MgSO_4_⋅7H_2_O | 1.5 g L^-1^ | 15 mg L^-1^ (S: 1.95 mg L^-1^) |
|  | KH_2_PO_4_ | 0.16 g L^-1^ | 1.6 mg L^-1^ (P: 0.36 mg L^-1^) |
| **2: Fe-EDTA** | FeCl_3_⋅6H_2_O | 64 mg L^-1^ | 64 μg L^-1^ (Fe: 13 μg L^-1^) |
|  | Na_2_EDTA⋅2H_2_O | 100 mg L^-1^ | 100 μg L^-1^ |
| **3: Trace elements** | H_3_BO_3_ | 185 mg L^-1^ | 185 μg L^-1^ (B: 32 μg L^-1^) |
|  | MnCl_2_⋅4H_2_O | 415 mg L^-1^ | 415 μg L^-1^ (Mn: 115 μg L^-1^) |
|  | ZnCl_2_ | 3 mg L^-1^ | 3 μg L^-1^ (Zn: 1.4 μg L^-1^) |
|  | CoCl_2_⋅6H_2_O | 1.5 mg L^-1^ | 1.5 μg L^-1^ (Co: 0.37 μg L^-1^) |
|  | CuCl_2_⋅2H_2_O | 0.01 mg L^-1^ | 0.01 μg L^-1^ (Cu: 3.7 ng L^-1^) |
|  | Na_2_MoO_4_⋅2H_2_O | 7 mg L^-1^ | 7 μg L^-1^ (Mo: 2.8 μg L^-1^) |
| **4: NaHCO_3_** | NaHCO_3_ | 50 g L^-1^ | 50 mg L^-1^ (C: 7.14 mg L^-1^) |


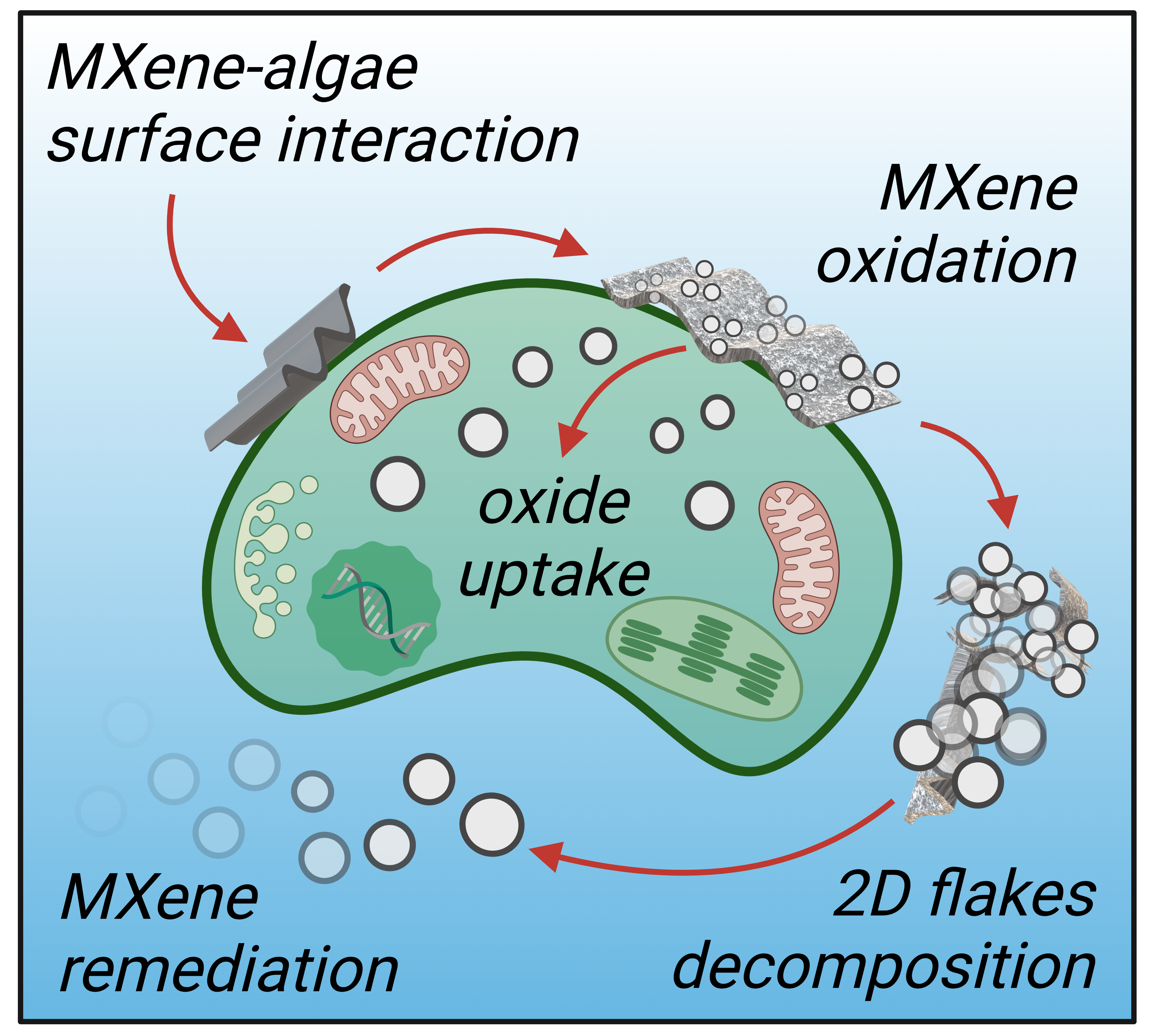


**Figure S26.** Schematic presentation of hypothesis for the MXene remediation with green microalgae *Raphidocelis subcapitata.* Created with BioRender.com

**References**

1. Halim J*, et al.* Electronic and optical characterization of 2D Ti2C and Nb2C (MXene) thin films. *Journal of Physics: Condensed Matter* **31**, 165301 (2019).

2. Halim J. An X-Ray Photoelectron Spectroscopy Study of Multilayered Transition Metal Carbides (MXenes). Drexel University (2016).

3. Rojas JV, Toro-Gonzalez M, Molina-Higgins MC, Castano CE. Facile radiolytic synthesis of ruthenium nanoparticles on graphene oxide and carbon nanotubes. *Materials Science and Engineering: B* **205**, 28-35 (2016).

4. Lei Y, Gao G, Liu W, Liu T, Yin Y. Synthesis of silver nanoparticles on surface-functionalized multi-walled carbon nanotubes by ultraviolet initiated photo-reduction method. *Applied Surface Science* **317**, 49-55 (2014).

5. Halim J*, et al.* X-ray photoelectron spectroscopy of select multi-layered transition metal carbides (MXenes). *Applied Surface Science* **362**, 406-417 (2016).

6. Echols IJ*, et al.* Oxidative Stability of Nbn+1CnTz MXenes. *The Journal of Physical Chemistry C* **125**, 13990-13996 (2021).
